# The functional organization of excitatory synaptic input to place cells

**DOI:** 10.1038/s41467-021-23829-y

**Published:** 2021-06-11

**Authors:** Michael D. Adoff, Jason R. Climer, Heydar Davoudi, Jonathan S. Marvin, Loren L. Looger, Daniel A. Dombeck

**Affiliations:** 1 Department of Neurobiology, Northwestern University, Evanston, IL USA; 2 Janelia Research Campus, Howard Hughes Medical Institute, Ashburn, VA USA

**Keywords:** Hippocampus, Spatial memory, Neural circuits, Neurotransmitters

## Abstract

Hippocampal place cells contribute to mammalian spatial navigation and memory formation. Numerous models have been proposed to explain the location-specific firing of this cognitive representation, but the pattern of excitatory synaptic input leading to place firing is unknown, leaving no synaptic-scale explanation of place coding. Here we used resonant scanning two-photon microscopy to establish the pattern of synaptic glutamate input received by CA1 place cells in behaving mice. During traversals of the somatic place field, we found increased excitatory dendritic input, mainly arising from inputs with spatial tuning overlapping the somatic field, and functional clustering of this input along the dendrites over ~10 µm. These results implicate increases in total excitatory input and co-activation of anatomically clustered synaptic input in place firing. Since they largely inherit their fields from upstream synaptic partners with similar fields, many CA1 place cells appear to be part of multi-brain-region cell assemblies forming representations of specific locations.

## Abstract (web summary)

Hippocampal place cells contribute to navigation and memory formation. Here, the authors use in vivo glutamate imaging to reveal patterns of excitatory input received by place cell dendrites and find more spatially tuned and functionally organized inputs arriving in the place field.

## Introduction

Hippocampal place cells encode an animal’s location in its environment through somatic action potential firing in discrete place fields^1^. Current models posit that these cells receive excitatory inputs with tuning curves that together tile all spatial environment locations, and that potentiating postsynaptic plasticity mechanisms select which inputs drive firing^2–8^. Some models also predict that the functional dendritic organization of excitatory input contributes to place firing through recruitment of supralinear dendritic summation^4,5,9,10^ or through synaptic plasticity induced by coactivation of anatomically clustered inputs^11–13^. However, the pattern of excitatory synaptic input leading to place field firing is currently unknown. Therefore, here we used resonant-scanning two-photon microscopy (2P) to record dendritic glutamate input to CA1 place cells of behaving mice with micron-scale spatial resolution using iGluSnFR^14^.

iGluSnFR is a membrane-targeted genetically encoded glutamate sensor that reports increases in extracellular glutamate concentration through increasing fluorescence in a manner independent of postsynaptic strength^15^. This indicator has previously been used in vivo to record excitatory synaptic input to dendrites of V1 neurons in a variety of mammalian species, including mice, ferrets, and primates^14,16–18^. iGluSnFR fluorescence transients caused by glutamate uncaging or afferent stimulation have been characterized in CA1 dendrites in slices and found to be useful as an optical reporter for single-spine quantal events^19^.

Using iGluSnFR in the hippocampus during spatial behaviors in virtual reality (VR), we found that the dendrites of individual place cells received significant excitatory input at track locations both inside and outside of the somatic place field. Many micron-scale dendritic regions of interest (ROIs) received highly spatially tuned excitatory input (place-ROIs), while other regions received input with little spatial tuning or no detectable input, and overall the ROIs were more spatially selective in place versus nonplace cells. The total excitatory input was greater in the somatic place field versus outside, and this increased excitation mainly arose from place-ROIs with spatial tuning overlapping the somatic field. Finally, excitatory input to place cell dendrites displayed functional clustering along the dendrite on the ~10 µm scale and this clustering was more pronounced in the somatic place field versus outside. These results implicate increases in total excitatory (glutamate) input and coactivation of anatomically clustered synaptic input in CA1 place field firing, the former result indicating that postsynaptic strength is not the sole determinant of the field location.

## Results

### Optical recording of excitatory input to CA1 neuron dendrites during spatial behaviors

**Fig. 1:**
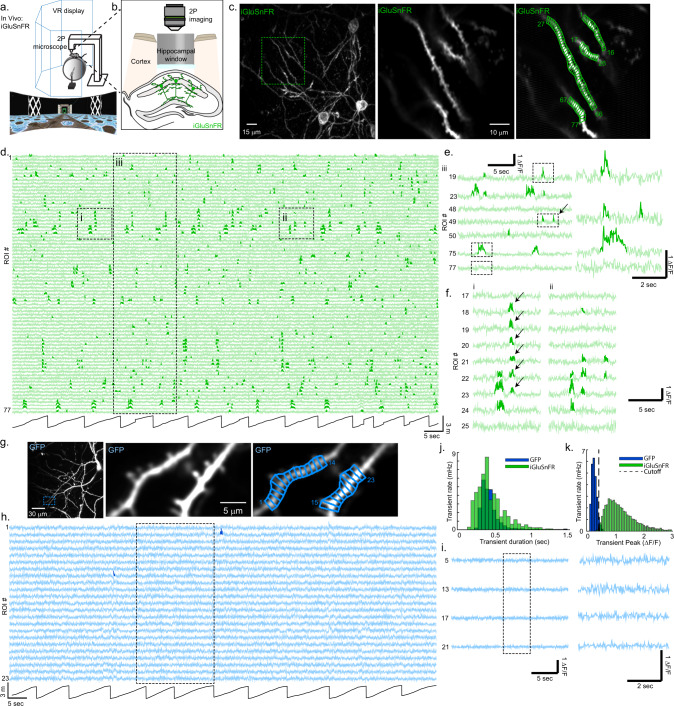
Optical recording of excitatory input to CA1 neuron dendrites during spatial behaviors. **a** Schematic of behavioral apparatus (top) and an example of the virtual linear track (bottom). **b** Schematic of hippocampal imaging window used to image CA1 pyramidal neurons expressing iGluSnFR with 2P microscopy during spatial behaviors in VR. **c** Left, example z-projection image of CA1 pyramidal neurons expressing iGluSnFR and imaged during behavior. Middle, mean image from time series acquired at a single imaging plane (from the region shown at the left). Right, same as middle, but with 77 1-µm ROIs shown in green. Similar results were obtained in 54 sessions from 11 mice. **d** iGluSnFR ΔF/F vs. time traces for each ROI shown in (**c**, right) acquired during linear track navigation (track position at the bottom, black). Significant transients highlighted in bold. **e** Left, expanded scale of a subset of traces shown in region iii from panel (**d**); arrow, example transients restricted to 1 ROI. Right, expanded scale of traces shown dashed at the left. **f** Left, expanded scale of traces shown in region i from panel (**d**) showing synchronous transients occurring over adjacent ROIs 18–23, marked with arrows. Right, expanded scale of traces shown in region ii from panel (**d**) showing transients in only some of the ROIs that were synchronously active in region i. Panel (**g**) same as panel (**c**), but neurons expressing GFP. Similar results were obtained in 13 sessions from 4 mice. Panel (**h**) same as panel (**d**), but from ROIs shown in panel (**g**, right). **i** Left, expanded scale of traces shown in the dashed region in panel (**h**). Right, expanded scale of traces shown dashed at the left. **j**, **k**. Histograms of durations (**j**) and peaks (**k**) of all significant iGluSnFR or GFP fluorescence transients from all 1 µm ROIs from all CA1 dendritic imaging sessions during behavior. Significant iGluSnFR transient amplitude cutoff threshold shown by a dashed line. GFP: *n* = 19 dendrites from 13 independent sessions from 4 mice. iGluSnFr: *n* = 109 dendrites from 54 independent sessions from 11 mice. Source data are provided as a Source Data file. VR virtual reality, 2P two-photon microscopy, ROI region of interest.

To optically record excitatory input to CA1 neuron dendrites during spatial behaviors, we sparsely labeled the CA1 pyramidal neuron population in adult mice with SF-iGluSnFR.A184S (iGluSnFR)^14^, installed a chronic hippocampal window, and used 2P microscopy to image the labeled neuron dendrites as mice performed spatial behaviors in virtual reality (VR, Fig. 1a, b). We recorded time-series movies from 109 basal and proximal oblique dendritic segments (mean ± SD: 126 ± 46 µm from soma, 2.2 ± 0.8 branch points from soma), from 47 fields of view ([40–72 µm] × [25–71 µm], 30–60 Hz frame rate) from 11 mice navigating in a familiar linear track for water rewards. Recordings from these dendrites are essential for understanding the synaptic basis of CA1 place field firing, since acute silencing of the presynaptic regions providing their inputs results in cessation of CA1 place firing^20^. We selected 1-µm length ROIs tiling the length of each dendritic segment and generated a ΔF/F vs. time trace for each ROI (Fig. 1c, d; each ROI treated separately). These traces consisted of numerous, statistically significant, positive-going iGluSnFR transients (see Methods, Fig. S1a; bold green in Fig. 1d), typically appearing as a sharp rise, followed by a slower decay to baseline, and with a range of amplitudes and durations (Fig. 1e, j, k; mean peak amplitude: 1.11 ± 0.65 (SD) ΔF/F; mean duration: 0.35 ± 0.21 (SD) seconds). iGluSnFR transients were typically restricted to 1 or 2 ROIs (76% restricted to 1 ROI, 91% restricted to 1 or 2 ROIs, Fig. 1d, e, arrow, Fig. S2a), though, rarely, synchronous transients occurring over larger numbers of adjacent ROIs, termed “large spatial extent transients” were also observed (3% of transients were ≥4 ROIs in spatial extent, Fig. 1f arrows, Fig. S2g, each ROI treated separately for these large spatial extent transients).

As a control, we repeated the above experiments and analyses but expressed the nonfunctional indicator GFP (19 basal and proximal oblique dendritic segments, *n* = 4 mice) instead of iGluSnFR. The GFP traces consisted of noise, with occasional false-positive transients occurring at the expected statistical rate for noise (Fig. 1g–i). The distribution of GFP noise transient amplitudes was smaller and almost completely nonoverlapping with the iGluSnFR transient amplitudes (Fig. 1k). Applying a minimum amplitude threshold of 0.40 ΔF/F left only 5% of GFP transients, but 98% of iGluSnFR transients; these remaining significant iGluSnFR transients were used for all subsequent analysis. Therefore, our methods allow for measurements of transient excitatory input to CA1 pyramidal neuron dendrites during spatial behaviors.

### Interpretation and characterization of iGluSnFR transients from CA1 neuron dendrites

While the original variant of iGluSnFR^21^ was previously characterized in hippocampal neurons in vitro^19,22^, the iGluSnFR variant used here (SF-iGluSnFR.A184S^14^) has not been similarly characterized. We therefore first characterized iGluSnFR responses to synaptic glutamate release using electrical stimulation of afferents in acute brain slices (see Methods) and 2P microscopy (Fig. S3a). We focused on dendritic regions that displayed clear all-or-none, stimulus-locked, evoked transients (significant transients; Fig. S3b–f). Previously, such stochastic responses during afferent stimulation have been attributed to the success or failure of glutamate release from a presynaptic terminal upon action potential arrival^19,23–25^. Evoked iGluSnFR fluorescence transients were highly localized along the dendrites, with increased change in fluorescence (ΔF/F) over ~1 µm (Fig. S3e, g). Based on the spatial extent and the stochastic nature of the evoked responses, we presume that these ΔF/F transients originate from glutamate released at single synapses, consistent with previous characterization^19^. iGluSnFR transient successes from these 1 µm dendritic regions of interest (ROIs) had mean peak amplitudes, durations, and integrals of 0.53 ± 0.27 (SD) ΔF/F, 0.36 ± 0.11 (SD) seconds, and 0.088 ± 0.047 (SD) ΔF/F sec, respectively (Fig. S3h, i). Applying the minimum 0.40 ΔF/F threshold used in vivo (Fig. 1k) to these slice iGluSnFR transients left 67% of the slice transients (Fig. S3h), indicating that the majority of these transients would be detected in vivo during behavior. We then used a second, separate, method to characterize iGluSnFR transients in a slice, with whole-cell recording and glutamate uncaging, and found similar results (Fig. S3k–v). Thus, the iGluSnFR signature of stimulation-evoked synaptic glutamate release is a short (~350 ms), highly localized (~1 µm) transient increase in fluorescence (~0.5 ΔF/F), indicating that iGluSnFR transients can be used as a proxy for synaptic glutamate release, reporting glutamate release occurring within a spatiotemporal window of ~1 µm and ~350 ms (Fig. S3j). Therefore, we reasoned that we could use iGluSnFR ΔF/F, averaged over 1 µm regions of CA1 pyramidal neuron dendrites, to provide a measure of total excitatory input received in these regions.

Multiple different spatiotemporal patterns of synaptic glutamate release could lead to the different amplitudes, durations, and spatial extents of iGluSnFR transients we observed in vivo. Here we describe what we consider the most likely patterns of release responsible for these in vivo observations; a schematic summary is shown in Fig. S4. The spatiotemporal profile of iGluSnFR ΔF/F characterized in a slice from axonal stimulation was highly similar to the smallest detected transients in vivo (Fig. S2c–f), indicating that this subset of in vivo transients is likely caused by similar single-synapse activations (presynaptic action potential; Fig. S4a). Consistent with this interpretation, most (91%) of the in vivo transients were highly localized (spatially restricted) on CA1 dendrites (76% within a single 1-µm ROI, 91% within 2 adjacent 1-µm ROIs, Fig. S2a), however, many were also larger in amplitude compared with the slice-afferent stimulation transients (Fig. 1k). This difference in amplitude was presumably caused by multiple synaptic activations within the 1-µm ROI within the decay time of iGluSnFR, resulting in summation (Fig. S4b). Since synaptic glutamate release was detected by iGluSnFR over ~1 µm dendritic regions (see Fig. S3e, g), and the fine CA1 dendrites investigated here have ~2–3 synapses/µm^26^, the transients from each 1-µm ROI likely report the integrated excitation received by a small number of synapses (i.e., iGluSnFR acts as a proxy for release within each ROI). Given the spatial and temporal sparsity of input, it is possible that many of the iGluSnFR transients from single ROIs in vivo are generated from single synaptic activations, with the other synapses in the ROI silent. It is also possible that some of these in vivo transients represent coactivation of multiple synapses within the ROI (Fig. S4b).

In contrast, the rare large spatial extent transients (Fig. 1f) are consistent with the synchronized release of glutamate from different presynaptic terminals onto multiple adjacent ROIs (Fig. S4c). This assessment is based on the following four observations: (1) in vivo iGluSnFR transient peak amplitude was not related to the spatial extent of the transient (Fig. S2b), (2) co-active adjacent ROIs recruited during large spatial extent transients could be recruited with fewer and/or non-adjacent ROIs within a short time of the large-extent transients (Figs. 1f, S2g), (3) large spatial extent transients had a plateau-like spatial profile (Figs. S4c, S5), and (4) minimal crosstalk was observed between nearby labeled dendrites when a large spatial extent transient occurred in one of the branches (Fig. S2h). Therefore, we used iGluSnFR transients in 1-µm ROIs as a proxy for the total integrated amount of synaptic glutamate release occurring within the region (referred to as excitatory input), localized transients (~1–2 ROIs) indicated activation of one or a few synapses, larger spatial extent transients were consistent with coactivation of more synapses over a larger region, and larger-amplitude/longer-duration transients were consistent with more synaptic activations, leading to more glutamate release and transient summation.

### Spatial tuning of excitatory synaptic inputs to place and nonplace cells

**Fig. 2:**
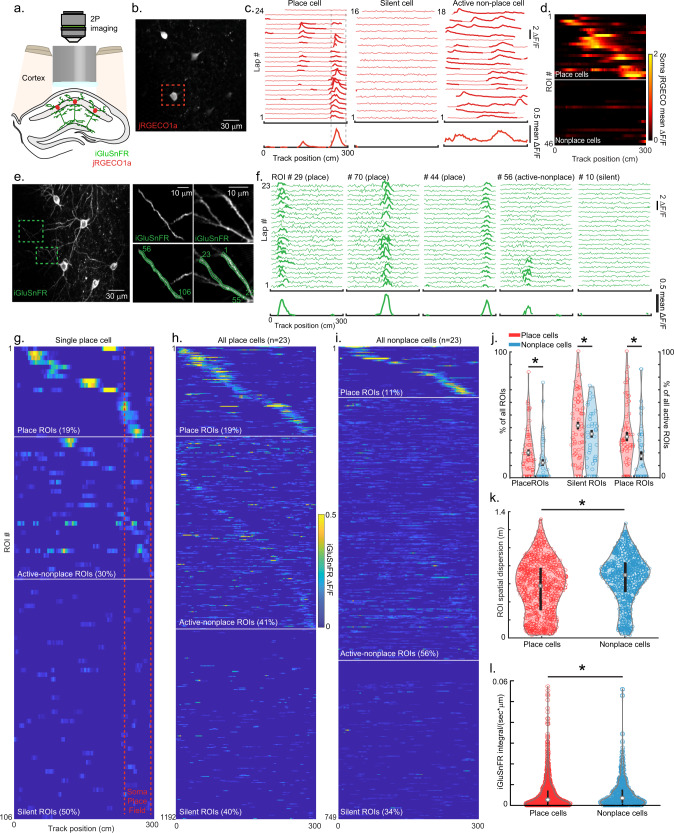
Spatial tuning of excitatory synaptic inputs to place and nonplace cells. **a** Schematic of experiments using 2P microscopy to image CA1 pyramidal neuron somatic firing patterns with jRGECO1a (red) and excitatory synaptic inputs to dendrites with iGluSnFR (green) during spatial behaviors in VR. **b** Example image of jRGECO1a fluorescence from labeled CA1 pyramidal neurons imaged during behavior. **c** Somatic jRGECO1a ΔF/F versus track position for each traversal of a single session (top) and mean ΔF/F versus position across all traversals (bottom) for three different neurons from different mice. Place cell at the left (cell highlighted in panel (**b**)) with place field track location between dashed lines, silent cell in the middle, and active–nonplace cell at the right. Significant transients highlighted in bold. **d** Mean somatic jRGECO1a ΔF/F versus track position across all traversals of a single session for all recorded neurons (each row represents single-neuron mean ΔF/F). Plotted via cross-validation within each cell category. **e** Left, example z-projection image of iGluSnFR fluorescence from labeled CA1 pyramidal neurons imaged during behavior (same neurons and field of view as shown in panel (**b**). Right, top, mean images from time series acquired at two different single-imaging planes (from regions shown at the left). Right, bottom, same as top, but with 106 1-µm ROIs shown in green. Similar results were obtained in 54 sessions from 11 mice. **f** iGluSnFR ΔF/F vs track position for each traversal of a single session (top) and mean ΔF/F versus position across all traversals (mean ROI map, bottom) for five example ROIs shown in panel(**e**, right), from place cell shown in panels (**b**, **c**). Significant transients highlighted in bold. Place-ROIs: 29, 44, 70; Silent-ROI: 10, Active–nonplace-ROI: 56. **g** Mean iGluSnFR ΔF/F versus track position across all traversals of a single session (mean ROI map) for all ROIs (each row represents a single ROI mean ΔF/F) shown in (**e**, right), from place cell shown in panels (**b**, **c**). Somatic place field track location between dashed lines. The percentage of ROIs in each ROI category also shown. Plotted via cross-validation within each ROI category. **h**, **i**. Same as panel (**g**), but for all ROIs from all 62 branches of all 23 place cells (**h**) or all 41 branches of all 23 nonplace cells (**i**). **j** Percentage of ROIs in each ROI category for place vs nonplace cells. Each circle represents a single branch. Mean ± bci across branches. (*p < 3.2e−3, likelihood ratio test, two-sided). *n* = 62 dendrites from 23 place cells from 35 independent sessions from 11 mice; *n* = 41 dendrites from 23 nonplace cells from 24 independent sessions from 11 mice. **k** Spatial dispersion of iGluSnFR transients in each ROI for all ROIs in place cells vs. nonplace cells (*p = 1.24e−4, likelihood ratio test, two-sided). **l** Mean amount of excitatory input per ROI per second (integral of all significant iGluSnFR transients in each ROI divided by recording time) for all ROIs in place cells vs. nonplace cells (*p = 9.1e−4, Rank-sum test, two-sided, place<nonplace). Source data are provided as a Source Data file for panels (**j**–**l**).

Many CA1 place cell models assume spatially tuned, Gaussian-shaped excitatory inputs^3,6,8^; however, recordings from the presynaptic CA2/3 populations have found neurons with a wide range of activity and spatial selectivity^27,28^. It is currently unknown which combination of these neurons provides excitation to CA1 place cells. To address this question, we sparsely labeled CA1 such that single pyramidal neurons co-expressed jRGECO1a (a red calcium indicator)^29^ and iGluSnFR (Fig. 2a). During track traversals, we first recorded somatic time-series movies (red channel, from 33 fields of view in 11 mice, 22.5 ± 1.5 (SE) traversals/somatic recording) as a measure of action potential firing and then adjusted the focal plane to record the excitatory input to the dendrites with iGluSnFR (green channel). Z-series morphology was used to trace dendrites back to the parent cell body (Fig. S6, see Methods). We identified 23 place cells (with 26 place fields) and 23 nonplace cells (including 16 active–nonplace cells and 7 silent cells, Fig. 2a–d) and recorded from their basal and proximal oblique dendritic segments (Place cell dendrites: 62 branches, 1192 µm of total length, 128 ± 44 µm (SD) from the soma, 2.4 ± 0.9 (SD) branch points from the soma, 17.6 ± 1.5 traversals/dendritic recording; Nonplace cell dendrites: 41 branches, 749 µm of total length, 124 ± 49 µm from the soma, 2.0 ± 0.6 branch points from the soma, 21.6 ± 1.8 traversals/dendritic recording; mean ± SE) (Fig. 2e). For each 1-µm dendritic ROI (each ROI treated separately), we plotted mean significant iGluSnFR transients (ΔF/F) versus track position over all traversals (mean ROI map) and classified three functional subtypes: ROIs with place fields (place-ROIs; mean place-ROI fields: 59.5 ± 14.5 cm (SD), Fig. S1d), ROIs without clearly defined fields (active–nonplace-ROIs), and ROIs without significant activity (silent-ROIs) (Fig. 2f). Individual place cells typically contained all three functional subtypes of ROIs (place-ROIs, active–nonplace-ROIs, and silent-ROIs), which covered the track with excitatory input (Fig. 2g). In some cells, mouse track position could be decoded based on this input (Fig. S1e–g). Interestingly, we found significantly more place-ROIs on basal dendrites than on oblique dendrites (17.2% vs 12.4%, χ_1_^2^ = 6.10, *p* = 0.014) and a small (${\beta }$ = 0.0040 µm^−1^), but significant (χ_1_^2^ = 8.5, *p* = 0.0036) increase in the probability that an ROI was classified as a place-ROI with increasing distance from the soma.

We then pooled the mean ROI maps across all place cells (Fig. 2h, plotted and sorted via cross-validation, see Methods) and separately from all nonplace cells (Fig. 2i) to examine the relative fraction of each ROI type in each cell type. We found that place cell dendrites contain 19 ± 2% (95% binomial confidence interval, bci) place-ROIs, 41 ± 3% (95% bci) active–nonplace-ROIs, and 40 ± 3% (95% bci) silent-ROIs, while nonplace cell dendrites contain 11 ± 2% (95% bci) place-ROIs, 56 ± 4% (95% bci) active–nonplace-ROIs, and 34 ± 3% (95% bci) silent-ROIs. Place cells contained a significantly larger percentage of place and silent-ROIs (χ_1_^2^ = 24.13, *p* = 8.9e−7 and χ_1_^2^ = 8.67, *p* = 3.2e−3, respectively, Likelihood ratio test, Fig. 2j), and a smaller percentage of active–nonplace-ROIs (χ_1_^2^ = 40.4, *p* = 2.1e−10 Fig. 2j), compared to nonplace cells (cell-by-cell comparisons in Fig. S1c). Interestingly, the pooled mean ROI maps (Fig. 2h, i) revealed that the active–nonplace-ROIs in place cells were more spatially selective than the active–nonplace-ROIs of nonplace cells (see the faint sequence in the active–nonplace-ROIs of place cells, not apparent in nonplace cells). Further, the track spatial dispersion of iGluSnFR transients in each ROI was significantly smaller on average in place cells versus nonplace cells (0.55 ± 0.011 m (SE) vs 0.65 ± 0.012 m (SE), respectively, *p* = 1.24e−4, rank-sum test, Fig. 2k, see Methods). Thus, place cells receive a combination of place, active–nonplace, and silent excitatory inputs that cover all track locations, and these inputs are more spatially selective in place versus nonplace cells.

To estimate the mean amount of excitatory input received by each cell type, we calculated the integral of all significant iGluSnFR transients per ROI per second and found that place cells receive 0.0054 ± 2.1e−4 ΔF/F, which was comparable but less (*p* = 9.1e−4, rank-sum test) than the 0.0058 ± 2.5e−4 ΔF/F received by nonplace cells (Fig. 2l). Therefore, on average, place cells receive slightly less excitatory input compared to nonplace cells per ROI per second.

Thus far, we have established that place cells receive a similar amount of mean excitatory input compared to nonplace cells, the excitation covers all track locations in both cell types, and excitatory inputs are more spatially selective in place versus nonplace cells. However, none of these findings explain the spatially selective firing of place cells. The following four (nonexclusive) hypotheses have previously been considered. First, postsynaptic plasticity mechanisms could potentiate the specific excitatory inputs that are preferentially active in-field^2,4,7–9^. Second, in-field disinhibition could allow for uniform excitation to drive track location-specific firing^30–32^. Third, total excitatory (glutamate) input could be greater in-field versus out-of-field^33–37^. Fourth, in-field active excitatory inputs might be more temporally co-active and anatomically clustered in the arbor versus out-of-field inputs, possibly making them more effective at driving firing through supralinear dendritic amplification^10,38–41^. Since our iGluSnFR measurements report total excitatory input and are insensitive to postsynaptic strength^15^ and inhibitory input, we could address the third and fourth hypotheses, but not the first and second.

### Total excitatory input is greater in the somatic place field versus out

**Fig. 3:**
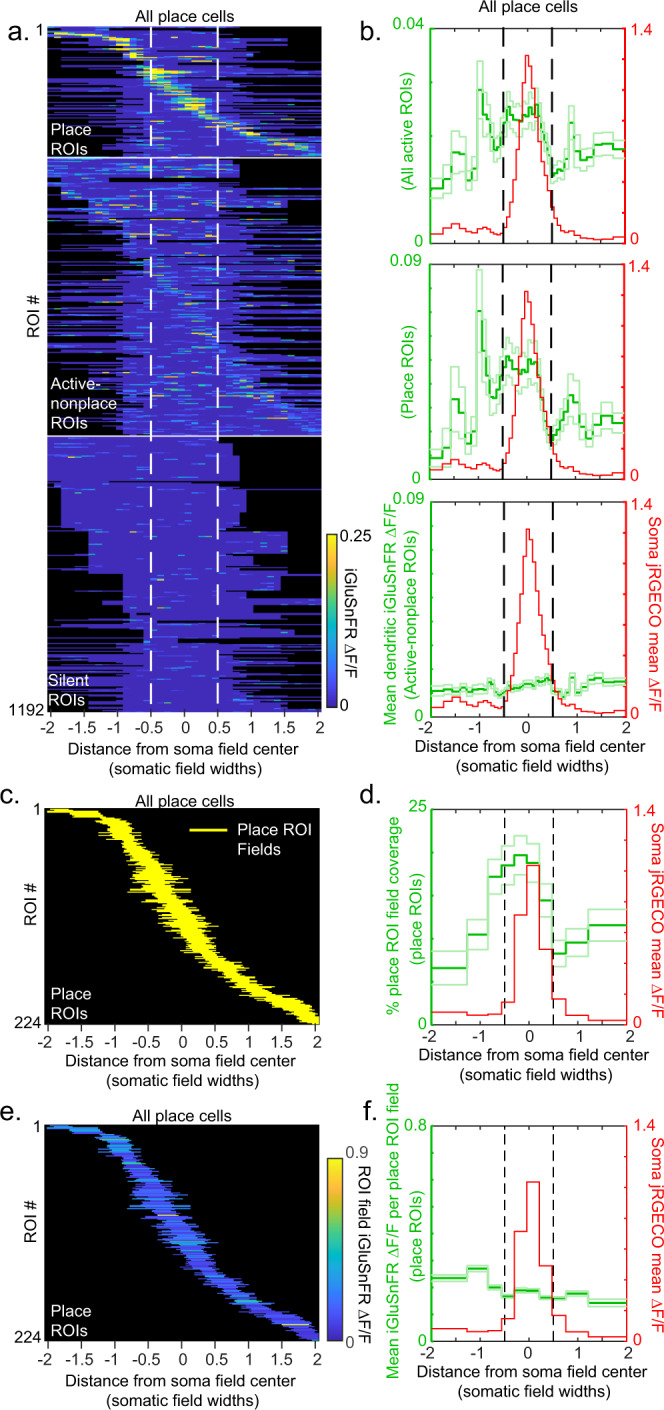
Total excitatory input is greater in the somatic place field versus out. **a** Mean iGluSnFR ΔF/F versus position from somatic place field center (in units of somatic place field width) across all traversals of a single session for all ROIs (each row represents single ROI mean ΔF/F) from all 62 branches of all 23 place cells (same ROIs as shown in Fig. 2h). Mean somatic place field between dashed lines. Plotted via cross-validation within each ROI category. **b** Mean total excitatory input (green) as a function of distance from the center of the mean somatic place field (red) for all active ROIs (top), all place-ROIs (middle), and all active–nonplace-ROIs (bottom). Mean (dark green) ± SE (light green). **c**, **d**. All place-ROI fields (yellow) versus position from somatic place field center (each yellow dash is a separate place-ROI field) (**c**) and percentage of place-ROIs with place-ROI field coverage (green) of binned positions from the center of mean somatic place field (red; (**d**), bin size different than in **b**). **e**, **f**. All place-ROI fields colored by their (mean) ROI field ΔF/F versus position from somatic place field center (**e**), and mean ΔF/F per place-ROI field (green) versus position from the center of mean somatic place field (red; (**f**), bin size different than in (**b**). Source data are provided as a Source Data file for **b**, **d**, and **f**.

To test whether the total excitatory input was greater in-field versus out-of-field (hypothesis 3), we plotted the mean ROI map for each ROI from each place cell (same data and same ROI order as shown in Fig. 2h) in units of somatic place field width and then pooled the maps together by centering each at the track location of the somatic place field peak (Fig. 3a, see Methods). By averaging over all active ROIs, we generated a plot of mean total excitatory input as a function of distance from the center of the mean somatic place field (Fig. 3b). This revealed that excitatory input was broadly increased around the somatic place field, with a shape and width reminiscent of the hill of depolarization observed from intracellular recordings^42^. In-field total excitation was significantly greater than out-of-field (in-field: 0.023 ± 0.002 (SE) ΔF/F, out-of-field: 0.017 ± 0.001 (SE) ΔF/F, *p* = 3.05e−10, rank-sum test on in-field vs out-of-field bins), which was also observed in the majority of individual fields (greater excitatory input inside versus outside of the somatic place field in 15 out of 26 place fields, ~60%; Fig. S7). Interestingly, when we averaged over place and active–nonplace-ROIs separately (Fig. 3b), we found that nearly all the in-field increase in excitatory input originated from the place-ROIs (place-ROIs in-field: 0.042 ± 0.004 (SE) ΔF/F, place-ROIs out of the field: 0.029 ± 0.003 (SE) ΔF/F, *p* = 4e−6, rank-sum test; active–nonplace-ROIs in-field: 0.014 ± 0.001 (SE) ΔF/F, active–nonplace-ROIs out-of-field: 0.012 ± 0.001 (SE) ΔF/F, *p* = 0.72, rank-sum test). Thus, place cells receive greater total excitatory input inside versus outside of the somatic place field (supporting hypothesis 3 above), and this increase originates mainly from the most spatially selective inputs.

We then tested whether this in-field total excitatory input increase was due to a greater percentage of ROIs active in-field or to increased excitatory input to in-field active ROIs. Since the in-field increase was mostly due to place-ROIs, we focused our analysis on the place-ROI fields (Fig. 3c–f). We plotted the percentage of place-ROI fields covering different positions from the center of the mean somatic place field and found the percentage was broadly increased around the somatic field (Fig. 3c, d, in-field 17.8 ± 1.4% (SE), significantly larger than out-of-field, 11.3 ± 0.9% (SE), *p* = 1.15e−05, rank-sum test), with an ~1.6x in-field to out-of-field percentage increase, similar to the total excitatory increase described above (Fig. 3b). Next, we plotted the mean ΔF/F per place-ROI field versus position from the center of the mean somatic place field and found that the mean in-field ΔF/F was not significantly different than out-of-field (Fig. 3e, f, in-field, 0.170 ± 0.01 (SE) ΔF/F, out-of-field, 0.172 ± 0.01 (SE) ΔF/F, *p* = 0.416, rank-sum test). Therefore, the in-field total excitatory input increase is largely due to an increased percentage (increased number) of place-ROIs with spatial tuning overlapping the somatic field versus outside of the field.

### Excitatory inputs are more temporally co-active and anatomically clustered in the somatic place field versus out

**Fig. 4:**
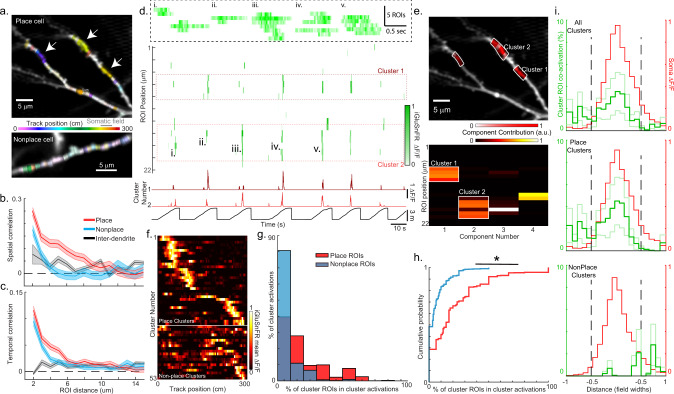
Excitatory inputs are more temporally co-active and anatomically clustered in the somatic place field versus out. **a** Dendritic segments from a place cell (top) and nonplace cell (bottom) with ROIs colored according to the center of mass of each ROI’s mean ΔF/F map. The brightness indicates peak ΔF/F value in the mean ΔF/F map divided by the spatial dispersion. Track location of somatic place field for place cell (top) shown in gray. Similar results were obtained from 23 place cells and 23 nonplace cells from 11 mice. **b** Spatial correlation (Pearson’s correlation) between the mean ΔF/F maps of all pairs of active ROIs on a single branch versus the dendritic distance between the pairs of ROIs, averaged over all branches from place (red) or nonplace (blue) cells. Inter-dendrite spatial correlation versus Euclidean distance for pairs of ROIs that belonged to different branches co-recorded in the same field averaged over all pairs of branches from all dendritic iGluSnFR recordings (black). Shaded regions, SEM. **c** Temporal correlation versus dendritic distance plots calculated as in panel (**b**), except significant transient-only traces were used instead of mean ΔF/F maps. Shaded regions, SEM. **d** Matrix of significant iGluSnFR ΔF/F transient vs time traces (green, 100 ms bins) for 22 neighboring ROIs from the top branch shown in panel (**a**, top) acquired during linear track navigation (track position at the bottom, black). ROIs for the 2 functional clusters from panel (**e**, bottom) outlined in red dashed boxes and the associated cluster ΔF/F vs time traces shown in red (bottom). The expanded time base for five events (i–v) shown in the black dashed box inset. Note that cluster activations (nonzero cluster ΔF/F) could occur as coactivation of all cluster ROIs, some of the cluster ROIs (often nonadjacent), or just one cluster ROI. **e** Bottom, non-negative independent components for the matrix shown in (**d**) (top), with 2 functional clusters highlighted in white. Top, Dendritic segments shown in (**a**), but ROIs belonging to 3 different functional clusters are highlighted in white. Similar results were obtained from 23 place cells and 23 nonplace cells from 11 mice. **f** Mean cluster iGluSnFR ΔF/F versus track position across all traversals of a single session for all functional clusters (each row represents single-cluster mean ΔF/F) from all place and nonplace cells. Plotted via cross-validation within each cluster category. **g** Histogram of percent of place (red) or active–nonplace (blue) ROIs that were part of a cluster that was co-active during a cluster activation (nonzero cluster ΔF/F). **h** Cumulative probability from histograms shown in panel (**g**). *p = 2.7e−117, Rank-sum test, two-sided. **i** Mean cluster ROI coactivation (green, percentage of cluster activations with > 70% cluster ROI coactivation) as a function of distance from the center of the mean somatic place field (red) for all clusters (top), all place clusters (middle), and all active–nonplace clusters (bottom). Mean (dark green) ± SE (light green). Source data are provided as a Source Data file for **b**, **c**, **g**, **h**, and **i**.

To test whether excitatory input inside versus outside of the place field was more temporally co-active and anatomically clustered in the arbor (hypothesis 4), we examined the functional dendritic organization of the ROIs in terms of both their spatial track selectivity and temporal activation patterns (Figs. 4, S8, and S9). We first examined intra-dendrite pairwise ROI spatial correlations (independent of ROI pair distance) and found significantly larger average correlations in place cells (0.060 ± 0.004) versus nonplace cells (0.017 ± 0.004, *p* = 1e−8, rank-sum test) or versus inter-dendrite correlations (0.025 ± 0.002, *p* = 4e−15, rank-sum test). When we colored ROIs according to their spatial selectivity, we often observed groups of adjacent ROIs (< ~10) with similar track selectivity (Fig. 4a, arrows), a pattern that appeared more often in place versus nonplace cells. Spatial correlation versus intra-dendrite ROI distance analysis (Fig. 4b) confirmed this observation and revealed elevated correlations on the ~10 µm scale in place cells (larger than nonplace cells, *p* = 3e−13, or inter-dendrite correlations, *p* = 7e−25, rank-sum test), with a slower correlation falloff in place cells versus nonplace cells (exponential fits, tau = 2.55 µm vs 1.43 µm, respectively, see also Fig. S9a–c) and no observable falloff for inter-dendrite ROI pairs (Fig. 4b), thus indicating functional anatomical organization of excitatory input to place cells on the 10 µm scale.

We then examined intra-dendrite pairwise ROI temporal correlations (independent of ROI pair distance) and found significantly larger average correlations in place cells (0.025 ± 0.001) versus nonplace cells (0.018 ± 0.001, *p* = 9e−10, rank-sum test) or versus inter-dendrite correlations (0.010 ± 0.0005, *p* = 2e−16, rank-sum test). Temporal correlation versus intra-dendrite ROI distance analysis (Fig. 4c) revealed elevated correlations on the ~10 µm scale in place cells (larger than inter-dendrite correlations, *p* = 7e−23, rank-sum test), with a slower correlation falloff in place cells versus nonplace cells (exponential fits, tau = 1.52 µm vs 1.14 µm, respectively) and no observable falloff for inter-dendrite ROI pairs (Fig. 4c). Further, a greater percentage of intra-dendrite ROIs within 10 µm of each other was likely to be co-active in place vs nonplace cells (Fig. S9d). Thus, excitatory input to place cell dendrites was functionally organized such that, on average, ROIs < ~10 µm apart encoded more similar track locations and were more likely to be co-active in time compared to ROIs at greater dendritic distances apart.

We then sought to determine whether the groups of nearby ROIs (~10 µm) with similar spatial tuning were also likely to be co-active in time in place cells. For each dendritic branch, we applied independent component analysis (see Methods) to the ROI iGluSnFR ΔF/F traces. We found that the ROIs making up the dominant components were often co-active in time and grouped in close proximity along the branch (Fig. 4d, e), though not necessarily adjacent; we defined such groups as functional clusters (see Methods; cluster size 4–9 ROIs, 4.5 ± 0.94 (SD) ROIs; 20.6 ± 20.7% (SD) of cluster ROIs co-active during any cluster activation; 39 clusters from 23 place cells; 0.90 ± 1.1 (SD) clusters/branch; 26.8% [95% bci 23.5–28.1%] of ROIs in place cells were in clusters, significantly greater than 5.9% [4.6%–6.5%] from shuffle distribution, χ_1_^2^ = 139.2, *p* ~ 0). Importantly, functional clusters and large spatial extent transients (synchronous transients occurring over ≥4 adjacent ROIs; Figs. 1f, S2g, and S5a, b, g, h) should not be equated directly with each other. Functional cluster activations (nonzero cluster ΔF/F) often occurred with the coactivation of only some of the cluster ROIs, which were often nonadjacent (Fig. 4d, g), though many large spatial extent transients were contained within the set of cluster activations. Cluster activations therefore occurred far more frequently (~30x) than large spatial extent transients (9.5e−3 ± 8.1e−3 SD vs 3.3e−4 ± 5.2e−4 SD activations/(µm s), respectively; rank-sum test *p* = 7.4e−25). We also searched for ROIs making up the dominant components that were co-active in time, but not grouped in close proximity along the branch. Such functional nonanatomical clusters were rare (three nonanatomical clusters across all place and nonplace cells) and were not considered further.

Interestingly, a greater percentage of place cell ROIs were in functional clusters versus nonplace cell ROIs (26.8% [bci 23.5–28.1%] vs 18.7% [bci 14.9–20.3%], 1.4x increase, LR test, ${\chi }_{1}^{2}$ = 8.1, *p* = 0.004). In both cell types, approximately 2/3 of clusters had place fields (place-clusters) and ~1/3 did not (active–nonplace clusters) (Fig. 4f), and during a cluster activation (nonzero cluster ΔF/F) place-ROIs that were part of the cluster were more co-active than active–nonplace-ROIs (*p* = 2.7e−117, rank-sum test, Fig. 4g, h). Place-ROIs were therefore more likely to be part of a cluster and co-active versus active–nonplace-ROIs (Fig. 4f–h). Importantly, in place cells, 44% (98/224) of place-ROIs were in clusters, which was greater than the 14.6% expected by chance (χ_1_^2^ = 57.5, *p* = 3.4e−14, likelihood ratio test). Thus, place cell dendrites contained clusters of nearby place-ROIs (< ~10 µm, nearly half of all place-ROIs) with similar spatial selectivity that was often co-active in time.

We then asked where cluster ROI activations occurred with respect to the somatic place field, and whether place– versus active–nonplace clusters differentially contributed to total in-field excitatory input. For place cells, we plotted the percent of time containing cluster ROI coactivations as a function of distance from the somatic place field (Fig. 4i). Interestingly, we observed a trend in which cluster ROIs displayed greater coactivation inside versus outside the field (2.0% inside vs 1.3% outside, similar shape as in Fig. 3b), and this in-field increase was mostly due to place-clusters (2.5% inside vs 1.4% outside). Similar results were found using a separate sliding window approach to define spatial clustered co-active ROIs (Fig. S9d, e). Therefore, clusters of nearby place-ROIs (< ~10 µm) with similar spatial tuning were more temporally co-active inside versus outside the somatic place field, consistent with the hypothesis (hypothesis 4 above) that supralinear dendritic amplification contributes to place field firing, but also consistent with place field formation models in which coactivation of anatomically clustered inputs preferentially induces synaptic plasticity^9,11–13^.

## Discussion

Here we established optical methods to record glutamatergic input received by CA1 place cell dendrites at 1 µm and 100 s of ms spatiotemporal scale in mice navigating along familiar linear tracks (Fig. 1). We found that dendrites of individual place cells received glutamate input at track locations both inside and outside the somatic place field^5,7,8,42^, with many micron-scale dendritic ROIs receiving highly spatially tuned excitatory input and others receiving input with little spatial tuning or no detectable input (Fig. 2f–h). On average, place cells received slightly less total excitation versus nonplace cells (Fig. 2l), but inputs to place cells were more spatially selective than those to nonplace cells (Fig. 2h, j, k). In place cells, we observed two general features of the excitatory input that suggested mechanisms of somatic place firing that were previously unknown: (1) greater total excitatory input inside versus outside of the somatic place field (Figs. 3, S7) and (2) more temporally co-active, dendritically clustered ROIs (< ~10 µm) inside versus outside the somatic place field (Figs. 4, S9).

Although iGluSnFR has proven useful for recording synaptic input to neurons during behavior^14,16–18^, it is critical to consider the limitations (and advantages) of the method and of slice calibrations when interpreting our datasets. First, since iGluSnFR is membrane-bound and detects changes in extracellular glutamate concentration, it reports only the synaptic input pattern, and not the total depolarization, which comes from the synaptic input pattern and the postsynaptic response to that input. Thus, iGluSnFR can provide information to deconstruct the excitatory input driving place firing into its different components (pre- vs post synaptic), which is difficult using other methods such as intracellular patch recording^5,42^ or dendritic calcium imaging^43^. Second, our slice and in vivo recordings indicate that synaptic activation leads to iGluSnFR transients extending ~1 µm and lasting hundreds of milliseconds. It is unclear whether the spatial extent of the transients indicates glutamate spillover from the synaptic cleft over this scale, which is similar to the scale of spillover described previously^44–47^, or is simply a reflection of our optical resolution, which is also ~1 µm. Either way, this measurement indicates that we are not able to resolve single synapses spatially and that synaptic input from multiple synapses is likely integrated within the 1 µm ROIs. Similarly, the temporal scale of iGluSnFR transients indicates that synchrony in synaptic input can only be defined down to the 100 s of ms scale. Given these considerations, iGluSnFR recording of synaptic glutamate release appears to be analogous to somatic calcium indicator recording of action potential firing (e.g., with GCaMP6f): somatic calcium transients act as a proxy for the underlying action potentials with transients reporting action potentials occurring within a spatiotemporal window of ~5–10 µm (cytoplasmic transients 5–10 µm from the membrane source) and ~100 s of ms (transient duration). In other words, somatic calcium transients are not a direct reporter of membrane potential changes, but a proxy signal of those changes. iGluSnFR transients may be analogous in that they are not a direct, sole reporter of cleft glutamate release but appear useful as a proxy signal of that release. Finally, while our slice calibration experiments (Fig. S3) demonstrate that iGluSnFR can report glutamate released by small amounts of presynaptic action potential firing or uncaging, the simultaneous postsynaptic response was not recorded (i.e., by patch recording or dendritic calcium imaging in low Mg^2+^). This makes it difficult to explicitly define our detection threshold and leaves open the possibility that some synaptic input could elicit a postsynaptic response, but not be detectable using iGluSnFR. It also even leaves open the possibility that some detectable iGluSnFR transients might not lead to a postsynaptic response if they arise from synaptic input to other, unlabeled neurons spilling over onto the labeled neuron (Fig. S4d), though our observation of a flat temporal correlation versus inter-dendrite ROI distance (Fig. 4c, black trace) down to ~2 microns (the closest pairs measured), suggests that this is unlikely down to at least this scale. Even considering these caveats, it seems unlikely that an increased detection efficiency, or reduced spillover detection, would change our major conclusions of increased excitatory input and temporally co-active, dendritically clustered ROIs inside versus outside of somatic place fields.

Excitatory input to the proximal dendrites recorded here provides most of the drive for CA1 place firing^20^, and here we showed that most of the in-field increase in total excitation was derived from place-ROIs with similar fields as the somatic field (Fig. 3b, middle). Thus, a significant amount of the CA1 place code in familiar environments appears to be inherited through integration of input from CA2/3 place cells with similar fields, rather than being formed through integration of nonspatial CA2/3 neurons. This suggests that many CA1 place cells are part of multi-brain-region cell assemblies forming representations of specific locations. Importantly, however, our results do not rule out additional (likely significant) contributions to place firing from other mechanisms or inputs, for example, from in-field disinhibition^30–32^, selective changes in postsynaptic strength^4,7–9^, from distal inputs^48^, or from different cellular properties such as those observed between superficial and deep CA1 pyramidal cells^49^.

We also found that the in-field total increase in excitatory input was due to a greater number of place-ROIs with spatial tuning overlapping the somatic field (Fig. 3c, d). This result suggests that place firing is driven in part by an increased number of place-tuned excitatory synapses releasing glutamate inside the somatic field versus outside. Such synaptic organization could be formed through genetically defined wiring^33,34,50^, new synapse formation to encode new environments^37^, and/or stimulus-dependent recruitment of prestrengthened neuronal ensembles formed through multiple iterations of synaptic plasticity and pruning^4,5,35,51,52^. Importantly, this result does not rule out the possibility that some of the increased in-field excitations come from increased (pre-) synaptic strength of in-field active synaptic inputs^36^. However, our results showed that place-ROIs with fields overlapping the somatic place field did not have increased iGluSnFR ΔF/F compared to those with fields outside the somatic field (Fig. 3e, f), indicating that increased presynaptic strength is not a major contributor.

We found that clusters of nearby place-ROIs (< ~10 µm) with similar spatial tuning were more temporally co-active inside versus outside the somatic place field (Fig. 4). While these functional cluster activations and large spatial extent transients cannot be equated directly with each other, many large spatial extent transients were contained within the set of cluster activations. We therefore presume that cluster activations in which only some of the cluster ROIs (often nonadjacent) were co-active were generated by the same mechanism (as large spatial extent transients), namely synchronized release of glutamate from different presynaptic terminals along the dendritic length (Figs. 1f, 4d, S2b, g, h, S5, and S8d). While large spatial extent transients contributed to increased correlations of many neighboring ROIs, they were not frequent enough to significantly contribute to the results seen in Figs. 2h, i, and 3b, since these results persisted even when large spatial extent transients were omitted from analysis (Fig. S10).

It is important to remember that iGluSnFR reports the synaptic input pattern (not the postsynaptic response to that input) with a temporal resolution of 100 s of ms. Therefore, this result provides an upper limit for measuring input synchrony (synchrony is < ~100 ms) that is entirely consistent with ms synchrony. Even still, since CA1 neuron dendrites contain voltage-gated channels and NMDA receptors that allow them to produce regenerative (spiking) events to amplify clustered and co-active (ms scale) synaptic inputs^10,38–40^, the presumed clustered coactivation of inputs observed here is consistent with the hypothesis that such supralinear summation contributes to place firing. This is consistent with previous in vivo calcium imaging demonstrating dendritic branch spiking during place firing^43^. The functional organization of excitatory input that we observed (Fig. 4) is also consistent with local learning rules strengthening local subsets of synapses to store memories, through LTP^4,9,10,12,13,40,53–57^, intracellular signaling^11,58,59^, or genetically defined clustered wiring^34,50^ and is seemingly more difficult to explain using only global plasticity mechanisms^7,8,60–62^. Finally, since context-specificity and global remapping drive much of the interest in place coding, future studies should aim to determine how the synaptic input properties described here change during place field formation.

## Methods

### Animals

In total 9–12 week old male C57BL/6 J (WT, Charles River) mice (20–30 g) were individually housed under a reverse 12 h light/dark cycle, in 40–60% humidity at 65–75° F. All experiments were approved and conducted in accordance with the Northwestern University Animal Care and Use Committee. Behavioral experiments were conducted during the animal’s dark cycle.

### Mouse surgery and virus injections

Mice were anesthetized (~1–2% isoflurane) and a small (~0.5–1.0 mm) craniotomy was made over the hippocampus (1.8 mm lateral, 2.3 mm caudal of Bregma). A low-titer Cre virus (AAV1-*CaMKII*-Cre, 1.51 × 10^8^ GC/mL, Addgene) was injected (1 injection of ~60 nL at a depth of ~1250 µm below the dural surface using a beveled glass micropipette: ~1–2 MΩ after beveling) in combination with a high titer of flexed-iGluSnFR.A184S virus^14^ (AAV2/1-*hSyn*-FLEX.SF-iGluSnFR.A184S, 5.87 × 10^12^ GC/mL) and flexed-jRGECO1a virus^29^ (AAV1-*hSyn*-FLEX.NES-jRGECO1a, 4.05 × 10^12^ GC/mL), leading to expression of SF-iGluSnFR.A184S and jRGECO1a in a sparse subset of the CA1 pyramidal neuron population. For control GFP imaging experiments, mice were injected following the same sparse labeling protocol, but with flexed GFP virus (AAV1-*CAG*-FLEX-EGFP-WPRE at 1.55 × 10^13^ GC/mL and AAV1-*CaMKII*-Cre at 1.51 × 10^8^ GC/mL). Mouse water scheduling began the day after virus injections (0.8–1.0 mL/day, and continued through all training and experiments) followed ~7 days later by a hippocampal window and head-plate implantation surgery^9,63^. For live slice imaging and glutamate uncaging experiments, mice were injected following the same sparse labeling protocol and allowed to recover for 3–4 weeks prior to hippocampal slice preparation.

### Behavior and training in virtual reality

We used the same virtual reality and treadmill setup as previously described^9^, consisting of a 1D treadmill (read with a rotary encoder using Labview 2011) and a view angle within the virtual environment straight down the track. Training in a 3 m virtual linear track began ~7 days after window implantation and continued until mice routinely ran along the track to achieve a high reward rate (> ~2 rewards/minute), rewards consisted of water (4 µL) delivered via a lick spout placed in front of the mice^9,63^. Mice were teleported back to the beginning of the track after each reward and after a 1 sec delay. Once this criterion was reached (~5–7 days of virtual reality training), imaging commenced.

### Two-photon imaging

A Moveable Objective Microscope (Sutter Instruments) was customized for our imaging experiments. The microscope consisted of a resonant scanning module (Vidrio), a 40×/0.80 NA water immersion objective (LUCPlanFL N, Olympus), and enhanced collection optics. Green iGluSnFR (or GFP) and red jRGECO1a fluorescence were routed to separate GaAsP PMTs (H10770PA-40) using a series of dichroic mirrors and band-pass filters (in order after leaving the back aperture; Semrock): FF665-Di02 long-pass dichroic, FF01-680/sp short-pass filter, FF560-Di01 long-pass dichroic, FF01-510/84 band-pass filter (Green), and 620/52 band-pass filter (red). Stray light from the virtual reality monitor was blocked using a custom box surrounding the top of the microscope objective and the overlying dichroic mirror (not including the tube lens, scan lens, galvos, or routing mirrors). This box had one hole on top, for entry of the excitation beam, which was covered with a color glass filter (FGL780, Thorlabs) and one hole on the bottom for the microscope objective. This bottom hole was sealed using the same loose black rubber tube and tight-fitting metal rings described previously^63^. ScanImage 2017 was used for microscope control and acquisition (Vidrio). Ti: Sapphire laser (Chameleon Ultra II, Coherent) light at 920 (for GCaMP6f, GFP) nm and fiber laser (Fidelity-2, Coherent) light at 1070 nm were used as the excitation sources for iGluSnFR and jRGECO1a, respectively. Laser average power at the sample (after the objective) was 47–136 mW (920 nm, mean: 65 mW) or 112–160 mW (1070 nm, mean: 138 mW). Pockels cells (350-105-02 KD*P, 302RM driver, Conoptics) were used to blank laser excitation at the edges of the field of view. Time-series movies (Somatic: 512 × 256–512 pixels, [214–281 µm] × [104–284 µm] field of view; dendritic: 512 × 256–512 pixels, ([40–72 µm] × [25–71 µm] field of view) were acquired at 30–60 Hz. A Digidata1440A (Molecular Devices) data acquisition system was used to record (Clampex 10.3) and synchronize position in the linear track, reward timing, and two-photon image frame timing.

Somatic red jRGECO1a fluorescence time series were acquired from the sparsely labeled cell bodies (5.6 ± 0.2 min/time series, 22.5 ± 1.5 track traversals/time series, mean ± SE). To maximize the number of place cells recorded, cells that were active along the track were targeted in most recordings, though other somas were often in the imaging field. The imaging plane and wavelengths were then adjusted to acquire dendritic green iGluSnFR fluorescence time series (4.7 ± 0.2 min/time-series, 19.8 ± 1.3 track traversals/time series, mean ± SE). It was possible online to follow dendrites from the targeted cell of interest to a more distal dendritic site, though offline analysis and tracing was later performed to confirm the parent cell body of the recorded dendrites. Dendritic recording planes were selected based only on dendritic branch morphology to obtain recordings from the longest dendritic branches possible, no online functional iGluSnFR measures were used to select branches. Other dendritic branches were often in the dendritic imaging field, and these dendrites were included in the cell-type-specific analysis (Figs. 2–4, S1, S4, and S7–9) when they could be traced to one of the somas recorded in the somatic time series (using z-series acquired at the end of the session, see below). Even if these other dendrites could not be traced to recorded somas, they were included in analysis that did not require somatic recordings (Figs. 1, S2, and S5). In a subset of recordings, after dendritic time-series acquisition, a second time-series recording was acquired from the soma to confirm the stability of the somatic spatial firing pattern. For control GFP recordings, no somatic recordings were made, but dendritic recording planes were selected using the same criteria as with iGluSnFR.

After time-series acquisition, z-series were acquired from each field of view from the external capsule fiber surface through the proximal apical dendrite (2 µm between planes) and motion-corrected using whole-frame cross-correlation^43^. All dendritic branches recorded during time series were traced offline in all 46 cells included here from motion-corrected z-series using Simple Neurite Tracer in Fiji (ImageJ). This method was used to identify the parent soma of each recorded dendrite by tracing them unambiguously back to the soma in the z-series. All dendritic distances from soma represent distance traveled along the neurite. Any ambiguous cases (i.e., crossing dendrites that could not be resolved) were not included in the cell-type-specific analysis and the dendrites were then only included in analysis that did not require somatic recordings (Figs. 1, S2, and S5).

### Data analysis

Imaging data were analyzed on a Dell Power Edge 720 Server using ImageJ (Version 1.47) and custom software written in MATLAB (r2018a). No statistical methods were used to predetermine sample sizes. Sample sizes were based on reliably measuring experimental parameters while remaining in compliance with ethical guidelines to minimize the number of animals used. Kolmogorov–Smirnov tests, *T*-tests, Shuffle tests, Likelihood ratio tests, and rank-sum tests were used to test for statistical significance when appropriate, and all statistical tests were two-sided, unless stated otherwise. No tests assumed normality, except the *T*-tests. Data collection and analysis were not performed blind to the conditions of the experiments. Data collection was not randomized. All data in the text and figures are labeled as mean ± std, mean ± sem, or mean ± bci. See Reporting Summary for more information.

### Image processing

Imaging data were analyzed on a Dell Power Edge 720 Server using ImageJ and custom software written in MATLAB r2018a. Motion correction of somatic plane jRGECO1a time series was performed using whole-frame cross-correlation^63^. Motion correction of dendritic plane iGluSnFR (or GFP) time series was performed using NoRMCorre^64^ and whole-frame cross-correlation^63^. Dendritic time series often required multiple rounds of motion correction (in series) to remove finer and finer motion. This was accomplished using different x and y shift values and/or different reference frames (typically an image built from averaging multiple frames) on each round. In some cases, the dendritic time series were cropped in the x and y dimensions around either single dendritic branches, or multiple dendritic branches in the same region of the field of view, and subsequent rounds of motion correction were performed on the cropped time series. Once dendritic movements were not reduced by further rounds of motion correction, the time series were visually inspected and either included if in-plane movements were small in comparison to the structures of interest (width of the dendrites) and out-of-plane (z) movement was minimal, or excluded if in-plane movements were larger than the structures of interest or if out-of-plane movement was visible. These criteria were strictly applied and were biased toward rejecting any borderline cases, ~2/3 of acquired dendritic time series were able to be sufficiently motion-corrected and included for further analysis. Thus, our inclusion criteria only focused on the structure (movement) of the dendritic branches and did not include any functional iGluSnFR measures. The same motion correction and inclusion criteria were applied to the GFP control data.

### ROI selection and fluorescent transient analysis

Somatic jRGECO1a time-series analysis: ROIs were selected by hand on the mean soma images to closely follow the outline of the soma. A background ROI to define the background fluorescence for each somatic ROI was drawn in a nearby dark region of the image. Fluorescence versus time traces were generated for each ROI by averaging the pixel values in each ROI in each frame. Each background ROI fluorescence trace was subtracted from its matching somatic ROI fluorescence trace (timepoint by timepoint). ΔF/F versus time traces were then generated for each background-subtracted somatic ROI trace similar to previous methods^63^. Briefly, slow changes in the fluorescence traces were removed by examining the distribution of fluorescence in a ±3 sec interval around each sample in the trace and normalized by the 8th-percentile value. Only periods of these traces when the mice were running along the track (velocity > 4 cm/sec, length of run periods> 40 cm) were included for further analysis. The baseline-corrected soma fluorescence traces (during track-running periods) were then subjected to the analysis of the ratio of positive- to- negative-going transients of various amplitudes and durations described previously^65^. We used this analysis to identify significant ΔF/F transients with a < 1% chance of being from noise sources (electrical noise, shot noise, and movement-induced noise), these identified significant transients were used in the subsequent analysis.

Dendritic iGluSnFR (and control GFP) time-series analysis: Dendritic ROIs from iGluSnFR or GFP-labeled dendrites were defined along each dendritic branch separately by first drawing an ROI by hand that closely followed the outline of the branch. About 1 µm segments along the dendritic branch (within the outline) were then identified using custom code. These segments defined the 1 µm ROIs (Figs. 1c, g, S3b, 2e, and 4a, Figs. S1e, S2h, and S5a, c, e) used for all subsequent dendritic analysis. Each ROI was treated separately, even for large spatial extent transients (e.g. a 10 µm length large-extent transient would be treated as 10 1-µm ROIs). A background ROI to define the background fluorescence for each dendritic branch was drawn in a nearby dark region of the image with size similar to the dendritic branch. Fluorescence versus time traces were generated for each ROI by averaging the pixel values in each ROI in each frame. Each background ROI fluorescence trace was subtracted from each 1 µm ROI fluorescence trace (timepoint by timepoint; background ROI from matching branch). The mean decrease in iGluSnFR fluorescence caused by bleaching in these traces was 15.9 ± 12.5% per 5 min of imaging. Such slow changes in the fluorescence traces were removed by examining the distribution of fluorescence in a ±1.5 sec interval around each sample in the trace and normalized by the 8th- percentile value. Only periods of these traces when the mice were running along the track (velocity > 4 cm/sec, length of run periods > 40 cm) were included for further analysis. Several exclusion criteria were then defined to handle ROIs with a weak fluorescence signal, ROIs with out-of-plane movements, and time periods in which large out-of-plane movements were observed across many ROIs: (1) ROIs at the ends of dendritic segments were not included for further analysis since they were most prone to out-of-plane movements. (2) ROIs with mean fluorescence of <1.5 counts were too dim for an accurate measure of ΔF/F and were excluded from further analysis; these criteria excluded 8.6% of ROIs. (3) Time periods (frames) in which large out-of-plane movements were observed across many ROIs (either across the same branch or different branches in the same field) were excluded from further analysis; these periods were identified by calculating the mean fluorescence versus time trace across all ROIs in a field, calculating the STD of this trace, and then excluding any time periods in which the fluorescence was >2STD from the mean in the positive direction or >1STD from the mean in the negative direction, this excluded 0.3% of frames during track running.

The remaining baseline-corrected dendritic ROI fluorescence traces were then subjected to the analysis of the ratio of positive- to- negative-going transients of various amplitudes and durations described previously^65^. When estimating baseline fluorescence (F) in this analysis, we used the positive-going noise above the mean F to measure noise std (keeping the original mean F in the std calculation to estimate std of the original trace) to avoid an underestimation of std caused by a floor effect. We used this analysis to identify significant ΔF/F transients with a <1% chance of being from noise sources (electrical noise, shot noise, and movement-induced noise), these identified significant transients were used in the subsequent analysis. ΔF/F versus time traces consisted of these significant transients with nonsignificant transient periods set to zero (significant transient-only traces). The above analysis was performed identically on the GFP- and iGluSnFR-labeled dendrites.

The GFP-significant transient-only traces consisted of noise, with only occasional noise transients occurring at the expected false-positive rate (Fig. 1g–i). The distribution of GFP noise-transient amplitudes was smaller (Fig. 1k) and almost completely nonoverlapped with the iGluSnFR transient amplitudes (significant transient-only traces). Applying a minimum amplitude threshold of 0.40 ΔF/F left only 5% of GFP transients, but 98% of iGluSnFR transients; these remaining significant iGluSnFR transients were used for all subsequent analysis and the small percentage (2%) of iGluSnFR transients less than the amplitude threshold were set to 0 in the significant transient-only traces.

Transient amplitude peak and duration (Fig. 1j, k) were characterized for every significant transient from each included ROI. The duration was defined as the full duration of the transient and the peak was defined as the peak ΔF/F within this duration. To estimate the mean amount of excitatory input received by the iGluSnFR-labeled dendrites in vivo, we calculated the integral (area under the transients) of the significant transient-only ΔF/F traces from all ROIs, and divided by the number of ROIs and the total recording time to arrive at the average integral per second per 1-µm ROI.

iGluSnFR transient event dendritic length and peak amplitude (Fig. S2a, b) were analyzed separately on each dendritic branch (only branches > = 8 ROIs in length were included; event-length measurements required different analysis compared to Fig. 1j, k). For each branch, we generated a matrix in which each row corresponded to a single ROI on the branch and the order of the rows corresponding to the position of the ROIs on the branch (i.e., neighboring rows corresponded to neighboring ROIs on the branch). The columns corresponded to 100 ms time bins, and the values of the matrix elements corresponded to average ΔF/F (from significant transient-only traces) over the 100 ms time bin. Transient events were defined as contiguous co-active (non-0 ΔF/F) ROIs. For transients restricted to 1 ROI, the length was defined as 1 µm and the peak amplitude as the peak ΔF/F value within the active time points. For transients extending to two or more ROIs, the length was defined by the time point(s) when the largest number of contiguous ROIs were co-active, and the peak amplitude was defined as the peak ΔF/F value (of any of the co-active ROIs) within those time points.

The mean image of iGluSnFR transients restricted to 1 µm ROIs (Fig. S2e) was generated by first identifying the time-series fluorescence (F) frame at which the transient peak occurred and excising a 4 µm by 4 µm region surrounding the transient pixels. This image was then transformed into a ΔF/F image on a pixel-by-pixel basis. For each pixel in the peak image, a fluorescence-versus-time trace was generated from the peak frame back to the preceding 0.6 s of the time series. This trace was normalized by the 8^th^ percentile of the pixel values (excluding the peak), and then the median of the resulting normalized trace was subtracted to arrive at a ΔF/F trace. The peak ΔF/F of this trace (the last time point) was assigned as the pixel value in the peak ΔF/F image. The ΔF/F images for each transient were then averaged together by first rotating each ΔF/F image to align the dendritic branch segment along the horizontal axis, and then calculating the center of mass of the ΔF/F image (center of mass of the image after thresholding ΔF/F > 0). To align the dendritic segments across images, if the site of the center of mass was below the mean horizontal axis of the dendrite, then the image was rotated 180°. The mean ΔF/F image was then generated by aligning the ΔF/F images on their center of masses and calculating the pixel-by-pixel mean ΔF/F value.

### Defining place and nonplace cells

Place cells were identified by first calculating the mean of the significant transient-only trace as a function of track position using 80 spatial bins (mean ΔF/F map, Fig. 2c, d) and then smoothing this mean ΔF/F map (3-bin boxcar). The original significant transient-only trace (vs. time) was then shuffled to randomize the transients with respect to tracking the position, and a mean ΔF/F map (smoothed using 3 bin boxcar) was generated for the shuffled trace. This was repeated 10,000 times to generate 10,000 mean-shuffled ΔF/F maps. We then identified significant spatial bins from the original mean ΔF/F map as bins in which ΔF/F values were greater than the ΔF/F values in 9500 of the 10,000 corresponding bins of mean-shuffled ΔF/F maps (*p* < 0.05). Place fields were identified as spatial regions that consisted of 5–46 contiguous significant spatial bins and displayed a significant transient within the region on >33% of track traversals. The bounds of significant fields were extended until the smoothed (3-bin boxcar) fluorescence map descended to 10% of the peak fluorescence or began to increase^66^. When place cells had more than one place field, each field was treated separately (20 place cells had 1 field, 3 had 2 or more fields). Nonplace cells were identified as any cells without a place field based on the above criteria, nonplace cells were further separated into silent cells and active–nonplace cells based on an activity threshold (silent: significant transients < 1% of time running on a track, active–nonplace: significant transients > 1% of time running on track). In total, 1192 1-µm ROIs were recorded from place cells and 749 1-µm ROIs were recorded in nonplace cells. These total lengths represent a significant fraction of the total length of proximal dendrites in a single CA1 pyramidal neuron^26,67^, and therefore by combining dendritic recordings over multiple cells based on cell type, we were able to generate a fairly complete description of the functional organization of excitatory synaptic input to an average place or nonplace cells.

In the majority of mice (7 out of 11 mice), both place and nonplace cells were recorded. Six out of 33 sessions included place and nonplace cells recorded simultaneously (same session).

### Defining place and nonplace-ROIs

Place-ROIs were identified by first calculating the mean of the significant transient-only trace as a function of track position using 80 spatial bins (mean ΔF/F map, Fig. 2f–i) and then smoothing this mean ΔF/F map (3-bin boxcar). The original significant transient-only trace (vs. time) was then shuffled to randomize the transients with respect to tracking the position, and a mean ΔF/F map (smoothed using 3-bin boxcar) was generated for the shuffled trace. This was repeated 1000 times to generate 1000 mean-shuffled ΔF/F maps. We then identified significant spatial bins from the original mean ΔF/F map as bins in which ΔF/F values were greater than the ΔF/F values in 950 of the 1000 corresponding bins of mean-shuffled ΔF/F maps (*p* < 0.05). Place-ROIs were identified as spatial regions that consisted of 5–46 contiguous significant spatial bins and displayed a significant transient within the region on >33% of track traversals. The bounds of significant fields were extended until the smoothed (3-bin boxcar) fluorescence map descended to 10% of the peak fluorescence or began to increase^66^. When place-ROIs had more than one place-ROI field, each field was treated separately (321 place-ROIs had 1 field, 9 had 2 or more fields). Nonplace-ROIs were identified as any ROI without a place field based on the above criteria; nonplace-ROIs were further separated into silent-ROIs and active–nonplace-ROIs based on an activity threshold (silent: significant transients < 1% of time running on track; active–nonplace: significant transients > 1% of time running on track).

### Fluorescence changes versus track position analysis

Mean ΔF/F maps were analyzed without smoothing. They were plotted via cross-validation (Figs. 2d, g–i, 3a, c, e, 4f, S1f and S10a, b,) by calculating the mean ΔF/F maps for the first half and second half of each session. The sorting order was determined by the peak ΔF/F value in the first-half map, and then the second-half map was displayed in the plots.

The spatial dispersion (Figs. 2k and 4a) of the significant transients of an ROI was defined based on the mean ΔF/F map as

1 $${\mathrm{{spatial}}}\,{\mathrm{{dispersion}}}=\frac{{\sum }_{i=1}^{N}{f}_{i}{(\mathrm COM-{\mathit x}_{i})}^{2}}{{\sum }_{i=1}^{N}{f}_{i}}$$

where i is spatial bin number, *N* is 80 spatial bins, ${f}_{i}$ is the ΔF/F value of the ith spatial bin, ${x}_{i}$ is the position of the ith spatial bin in cm, and COM is the center of mass of the mean ΔF/F map in cm and is defined as

2 $${\rm{COM}}=\frac{{\sum }_{i=1}^{N}{f}_{i}{x}_{i}}{{\sum }_{i=1}^{N}{f}_{i}}$$

Mean ΔF/F maps in units of somatic place field width (Fig. 3a) were generated by scaling the distance axis in the mean ΔF/F maps by the width of the somatic place field (full width), and then pooling the maps together by centering each at the track location of the somatic place field peak. For place cells with more than 1 place field, mean ΔF/F maps for each field included the track locations before and after the field up to either the edge of the next place field or the track edge (whichever came first). Place fields with one edge at a track edge were included, and the width of these fields was defined as twice the distance from the place field peak to the field edge (field edge not at track edge). Since the mean ROI maps for each ROI were plotted by centering each at the track location of the somatic place field peak, there are distances from the somatic place field for each ROI for which there were no data, these regions were plotted as black in Fig. 3a. For example, if the somatic place field associated with an ROI was toward the right side of the track, then when the mean ROI ΔF/F is plotted centered on the somatic field, there are no data to the right of the field center since those locations are past the right end of the track.

The plots of mean total excitatory input as a function of distance from mean somatic place field center (Fig. 3b) were generated by calculating the average ΔF/F across all place and active–nonplace-ROIs in each spatial bin (including only spatial bins for each ROI that contained ΔF/F values). Spatial bin size varied as a function of distance from the somatic field center so that each bin contained a similar number of datapoints (i.e., larger bins further from field center).

The plot of the percentage of place-ROIs with place-ROI field coverage of binned positions from the center of the mean somatic place field (Fig. 3c, d) was generated by calculating the percentage of place-ROIs with an ROI field in each spatial bin (including only spatial bins for each place-ROI that contained ΔF/F values). The plot of mean ΔF/F per place-ROI field versus position from the center of the mean somatic place field (Fig. 3e, f) was generated by first assigning all track locations in each place-ROI field the mean ΔF/F value over the field and then calculating the average of these mean-field ΔF/F values across all place-ROI fields in each spatial bin (including only place-ROI fields). Spatial bin size varied as a function of distance from somatic field center for both plots (Fig. 3c–f), so that each bin contained a similar number of datapoints (i.e., larger bins further from field center).

### Bayesian decoding

The Bayesian decoder used in Fig. S1e–g was adapted from a previously described method^68^. Decoding was performed on the likelihood that a significant transient occurred in a time frame, trained on the first 80% of the session, and tested on the last 20%. The session was divided into Δ*t* = 0.5 s bins. The conditional likelihood that an animal is in position ${x}_{i}$ given the number of active frames during a time window ($n$) is

3 $$p\left({x}_{i},|,n\right)={p}_{X}\left({x}_{i}\right)\left(\mathop{\prod }\limits_{j=1}^{M}{f}_{i,j}^{{n}_{j}}\right){e}^{-\Delta t\mathop{\sum }\nolimits_{j=1}^{M}{f}_{i,j}}$$

where ${p}_{X}\left({x}_{i}\right)$ is the (marginal) probability that the animal is in the ${i}{{th}}$ spatial bin during a time sample, ${f}_{i,j}$ is the average rate of significant frames by the ${j}{{th}}$ ROI in the ${i}{{th}}$ spatial bin, ${n}_{j}$ is the number of significant frames observed during the time window in ROI $j$, and $M$ is the total number of ROIs. The decoded position was selected as the one with maximum conditional likelihood.

### Functional dendritic organization analysis

Images in Fig. 4a were generated by coloring each ROI according to the center of mass of its mean ΔF/F map. The brightness of each ROI was defined by the peak ΔF/F value in the mean ΔF/F map divided by the spatial dispersion.

Spatial correlation versus distance (Fig. 4b) was calculated as the Pearson’s correlation between the mean ΔF/F maps of all pairs of active ROIs (excluding silent-ROIs) > 1 µm apart on a single branch versus the dendritic distance between the pairs of ROIs. Average spatial correlation versus distance plots was generated by averaging over all pairs belonging to place or nonplace cells. Inter-dendrite spatial correlation versus distance was calculated in the same way, except pairs of ROIs belonged to different branches that were co-recorded in the same field, and distance was defined as the Euclidean distance between the ROIs. Temporal correlations (Fig. 4c) were calculated in the same way, except the significant transient-only traces were used instead of mean ΔF/F maps. Spatial and temporal correlations independent of ROI distance were calculated in the same way (ROIs >  1 µm apart and excluding silent-ROIs), but without binning the ROI pairs by distance. To compare the falloffs in correlations in Fig. 4b, c, we fit the place cell, nonplace cell, and inter-dendrite correlation versus ROI distance traces with an exponential decay. We then compared this to the nested model where place cell, nonplace cell, and inter-dendrite traces shared a decay time constant. Log-likelihoods were calculated assuming stationary Gaussian noise and compared with a likelihood ratio test.

Sliding-window ROI coactivation analysis (Fig. S9d, e) was performed separately on each dendritic branch (only branches > 10 ROIs were included). For each branch, we generated a matrix in which each row corresponded to a single ROI on the branch and the order of the rows corresponded to the position of the ROIs on the branch (i.e., neighboring rows corresponded to neighboring ROIs on the branch). The columns corresponded to 100 ms time-bins, and the values of the matrix elements corresponded to average ΔF/F (from significant transient-only traces) over the 100 ms time-bin. The number of co-active ROIs within a window of 10 rows × 1 column was quantified over all possible positions of the window in the matrix. For place versus nonplace cells (Fig. S9d), the resulting histograms of co-active ROIs over all window positions were pooled by cell type. For place versus nonplace-ROIs in the somatic field versus out of somatic field measurements (Fig. S9e), window positions inside versus outside of the somatic field were pooled separately and only including either place-ROIs or nonplace-ROIs. At least one of the ROI type of interest was required to be present in a given window to be included.

Functional clusters (Fig. 4d–i) were defined separately on each dendritic branch (only branches > 8 active ROIs were included). For each branch, we generated a matrix in which each row corresponded to a single ROI on the branch and the order of the rows corresponded to the position of the ROIs on the branch (i.e., neighboring rows corresponded to neighboring ROIs on the branch). The columns corresponded to 100 ms time bins, and the values of the matrix elements corresponded to average ΔF/F (from significant transient-only traces) over the 100 ms time bin. We then decomposed the matrix into components by reconstructing ICA using PCA as an initial guess. The resulting weight matrix was then made nonnegative by finding the closest nonnegative matrix as measured by the Frobenius norm. The rows in the resulting weight matrix corresponded to the ROI number, the columns to independent components, and the values of the matrix elements to the contribution of each ROI to each component. Neighboring rows corresponded to neighboring ROIs on the branch, although notably the order of rows has no effect on the PCA/ICA analysis. Within each component, we normalized the component contributions by the peak ROI component value and then identified all ROIs with values > 20% of the peak. Among these highest-contributing ROIs, we then defined functional clusters as groups of at least 4 ROIs that were in close proximity. All ROIs in the groups of > = 4 ROIs were required to be adjacent, with the exception that a gap of 1 ROI between the contributing ROIs was allowed and at least 70% of the ROIs in the functional cluster were required to be contributing ROIs (values > 20% of the component peak). The ROI order (branch location) was then shuffled 1,000 times and, for each shuffle, clusters were identified using the above criteria. Across all dendritic branches, the number of clusters from the original (unshuffled) dataset exceeded the number of clusters found in all 1000 shuffles (Original versus shuffle: ${\chi }_{1}^{2}$ = 136.2, *p* ~ 0; Place cells original versus shuffle: ${\chi }_{1}^{2}$ = 139.2, *p* ~ 0; Nonplace cells original versus shuffle: ${\chi }_{1}^{2}$ = 43.4, *p* = 4.56e−11). For the original and shuffle data, all possible numbers of components were analyzed up to one-half the number of active ROIs on the branch, so long as the number of detected clusters was nondecreasing, and any detected clusters within these components were used for further analysis. Importantly, the results shown in Fig. 4g, h, i are robust to changes in the 2 most important thresholds used in this analysis (the ROI contribution threshold—20% value used and the cluster coactivation threshold used in Fig. 4i—70% value used), which is shown in Fig. S8. Note that the average number of ROIs recorded per place cell (19.2 ± 12.4 ROIs) was not significantly larger than the average number recorded per nonplace cell (18.1 ± 12.3 ROIs) (*p* = 0.60, rank-sum test) and therefore the average number of ROIs per cell did not bias cluster analysis between place and nonplace cells.

Cluster ΔF/F traces in time (Fig. 4d) consisted of the weighted sum of the iGluSnFR ΔF/F traces of the ROI in each cluster, with weighting determined by each ROIs non-negative independent component contribution. Mean cluster ΔF/F maps were generated by calculating the mean cluster ΔF/F traces as a function of track position over all traversals using 80 spatial bins; these cluster maps were plotted via cross-validation (Fig. 4f), as described above (see “Fluorescence changes versus track position analysis” section). Place and nonplace clusters were defined using the same methods as described above for individual ROIs (see “Defining place and nonplace-ROIs” section). Cluster activation was defined as anytime a cluster component trace was >0, that is, whenever any ROI in the cluster was active. The percent of cluster ROIs (place or active–nonplace-ROIs) that were co-active in each cluster activation (Fig. 4g) was defined as the fraction of (place or active–nonplace) ROIs active during any cluster activation. Plots of cluster coactivation vs distance from the mean somatic field (Fig. 4i) were made by making spatial bins in units of somatic field widths, and then calculating the percentage of time during cluster activations in which >70% of cluster ROIs were co-activated. Spatial bin size varied as a function of distance from somatic field center so that each bin contained the same amount of data (i.e., larger bins further from field center).

To search for functional non-anatomical clusters, temporally clustered, spatially disperse activity was examined as described above for functional clusters. Isolated ROIs were identified as spatial groups between 1 and 3 ROIs as above, with values > 20% of the peak and a gap of 1 ROI between contributing ROIs allowed. We then searched for components that contained > = 4 such isolated ROI groups (> = 2 ROIs between contributing ROIs) as spatially disperse, temporally clustered components. Across all components (from all place and nonplace cells), we only identified three such functional nonanatomical clusters, suggesting that such clustering is rare on the scale of the dendritic segments recorded here.

### Hippocampal slice preparation

Transverse hippocampal slices (~300 μm) were prepared from iGluSnFR virus-injected male C57BL/6 J mice (same-age mice and same injection procedures as used for in vivo experiments; see “Mouse surgery and virus injections” section above) using a vibrating microtome (VT1200S; Leica Systems, Germany). For GCaMP6 imaging and uncaging experiments, the same virus injection procedures were used as described for iGluSnFR, but AAV1-*hSyn*-flex-GCaMP6f or s, (1.4 × 10^13^ GC/mL) was used instead. Animals were anesthetized with isoflurane and perfused with ice-cold sucrose artificial cerebrospinal fluid (ACSF) solution containing (in mM) 85 NaCl, 2.5 KCl, 1.25 NaH_2_PO_4_, 25 NaHCO_3_, 25 glucose, 75 sucrose, 0.5 CaCl_2_, and 4 MgCl_2_, 3 sodium pyruvate, and 1 ascorbic acid, saturated with 95% O_2_ and 5% CO_2_. After slices were cut (in the same sucrose ACSF used for perfusion), they were transferred to a warm (32 °C) incubation chamber with oxygen-bubbled ACSF containing (in mM) 125 NaCl, 2.5 KCl, 25 NaHCO_3_, 1.25 NaH_2_PO_4_, 1 MgCl_2_, 2 CaCl_2_, and 25 glucose, for 25 min after which time they were allowed to recover at room temperature in oxygenated ACSF for 1 h before imaging and/or intracellular patch recording.

### Glutamate uncaging: slice imaging, intracellular recording, and glutamate uncaging parameters

Glutamate uncaging and imaging of hippocampal CA1 pyramidal neuron basal and proximal oblique dendrites in vitro were performed on an Ultima two-photon laser scanning microscope (Bruker, former Prairie Technologies, Middleton, WI) equipped with dual galvanometers driving two Ti: Sapphire lasers (Chameleon, Coherent). The lasers were tuned to 920 nm for iGluSnFR imaging Figs. S3o–v, S5c–f, or for GCaMP6 imaging, Fig. S3w, x) imaging or 840 nm for Alexa 594 imaging, and 720 nm for glutamate uncaging, and the intensity of each laser was independently controlled with electro-optical modulators (Conoptics). Imaging and uncaging were performed with an upright Zeiss Axiovert microscope using a 40x, 1.0 numerical aperture water immersion objective. During imaging and uncaging, slices were maintained at a constant temperature ranging from 30–34 °C (mean 32.4 °C and bathed in recirculating oxygen-bubbled ACSF containing 3 mM MNI-caged L-glutamate (4-methoxy-7-nitroindolinyl-caged L-glutamate, Tocris), 1 μM TTX (Tocris Bioscience), and 2 μM of GABA_A_ receptor antagonist SR-95531 (Tocris Bioscience); in a subset of GCaMP6 uncaging and imaging experiments, Mg^2+^ was not included in the ACSF (0 Mg^2+^, Fig. S3x). MNI glutamate was uncaged using 500 μs pulses (10–70 mW after the objective) with a 120 μs interstimulus interval for multisite stimulation (Figs. S3, S5c–f). All dendritic branches were 25–75 μm from the slice surface. Intracellular patch recordings were made using patch electrodes (3–6 MΩ) filled with intracellular solution containing the following (in mM): 115 K-gluconate, 20 KCl, 10 HEPES, 10 Na_2_ creatine phosphate, 2 Mg-ATP, and 0.3 Na-GTP. For uncaging experiments without iGluSnFR (Fig. S3k–m), 0.025 mM Alexa 594 was included in the pipette. Time-series movies were acquired at >15 Hz (mean 43.5 Hz) for the duration of uncaging events and were analyzed with MATLAB (MathWorks) and ImageJ following motion correction (see “Slice ROI selection and fluorescent transient analysis” section).

### Glutamate uncaging analysis

Slice time-series movies were motion-corrected using whole-frame cross-correlation to remove any slow drift. About1 μm ROI selection and generation of significant transient-only traces for each ROI were performed using the same procedure as used for in vivo imaging dendritic datasets (see “ROI selection and fluorescent transient analysis” section). iGluSnFR transient amplitude peak (Fig. S3r), duration (Fig. S3s), and integral (Fig. S3t) were characterized for every significant transient generated by single 25–29 mW uncaging stimulations—96% (43/45) of these stimulations generated significant transients. For peak and duration, if significant transients were generated in more than 1 ROI, the ROI with the largest amplitude was used for analysis. The duration was defined as the full duration of the transient and the peak was defined as the peak ΔF/F within this duration. For the integral of the transients generated by single 25–29 mW uncaging stimulations, we calculated the integral of all significant transients (i.e., if significant transients were generated in more than 1 ROI, the integral included all responding ROIs).

### Afferent stimulation: slice imaging and stimulation parameters

For afferent stimulation experiments (Figs. S3a–j, S2d, f) 2P microscopy (Ultima two-photon laser scanning microscope, 920 nm excitation, 40x, 1.0 numerical aperture water immersion objective) was used to identify a CA1 dendrite (mostly basal, with a few recordings from oblique dendrites) of interest that was connected to a parent CA1 cell body. Slices were maintained at 30–34 °C in oxygen-bubbled ACSF. Electrical stimuli were then delivered via a theta-glass pipette positioned using DODT contrast imaging in stratum oriens or stratum radiatum in close proximity (~10–50 µm) to the iGluSnFR-labeled dendrite of interest. Stimulation electrodes were filled with ACSF alone or ACSF+AlexaFluor 594 (50 µM) for improved visualization. To identify dendritic regions with electrical stimulus-evoked fluorescence transients, we used paired-pulse stimulation (50–250 µA, 0.1 ms pulses, 50 ms interpulse interval). Once a responsive dendritic region was identified, single-pulse stimulation (20–250 µA, 0.1 ms pulses) was used and was gradually reduced to the minimal current that still evoked time-locked transients. 2P microscopy time-series movies of the responsive dendritic branch were acquired at 15–58 Hz during repeated application of this current (6–12 stimuli at 0.5–1 Hz).

### Afferent stimulation analysis

Time-series movies were motion-corrected as described above (Methods: Image processing). One-µm length ROIs tiling the length of each dendritic segment were selected and ΔF/F versus time traces were generated for each ROI as described above (Methods: ROI selection and fluorescent transient analysis). Significant ΔF/F transients (with <1% chance of being from noise sources: electrical noise, shot noise, and movement-induced noise) were then identified as described above (Methods: ROI selection and fluorescent transient analysis). Electrical stimuli with time-locked significant ΔF/F transients were considered stimulus successes, and stimuli with no significant ΔF/F transient detected were considered stimulus failures. To be considered for further analysis, 1-µm ROIs had to meet the following criteria: (1) baseline ΔF/F noise level STD <0.3 ΔF/F (mean noise 0.17 +/− 0.09 ΔF/F closely matching our in vivo baseline noise level of 0.19 ± 0.09), (2) significant transient successes were observed at <85% of electrical stimuli (mean percentage of stimuli with significant transient success: 22 ±15%), and (3) since some spontaneous significant transients were observed, more than half of all significant transients had to be time-locked to electrical stimuli. To identify ROIs with all-or-none evoked transients and to minimize the possibility of detecting spillover from nearby synapses (onto the labeled dendrite or nearby unlabeled dendrites), the ROIs further had to meet the following criteria: (1) for all stimulus failures in an ROI, no detectable increase in fluorescence was observed after the stimuli in the stimulus-triggered average of ΔF/F (*t*-test between the ΔF/F values 300 ms before the stimulus vs 300 ms after the stimulus, *p* > 0.2, highly similar results were obtained when the *t*-test was performed on individual stimulus failures and including the ROI only if no detectable fluorescence increase was observed for any stimulus failure (*p* > 0.2)), (2) for all stimuli resulting in stimulus failures in an ROI, stimulus successes were observed at <10% of the stimuli in the neighboring ROIs (the two neighboring ROIs), and (3) for all stimuli resulting in stimulus successes in an ROI, neighboring ROIs were allowed to have stimulus successes, which often occurred when the stimulated synapse of interest was bisected by the ROIs. ROIs meeting these criteria were used for all analysis shown in Figs. S3a–j, S2d, f. The median ΔF/F images of stimulus successes and failures (Fig. S3g) were generated as described above (Methods: ROI selection and fluorescent transient analysis) using the median ΔF/F values instead of mean. Note that the ~1 µm extent of the iGluSnFR transients is the same size as the expected point spread function; therefore, this size should be considered an upper limit to the detected spread of glutamate beyond the synapse; the actual spread may be <1 µm.

### Reporting summary

Further information on research design is available in the Nature Research Reporting Summary linked to this article.

## Supplementary information

Supplementary Information

Peer Review File

Reporting Summary

## Data Availability

The data that support the findings of this study are available from the corresponding author upon reasonable request. Source data are provided with this paper.

## Source data

Source Data

## Code availability

The custom code that supports the findings of this study is available from the corresponding author upon reasonable request.
