# Supplementary material for: The functional organization of excitatory synaptic input to place cells
Source: Nat Commun. 2021 Jun 11;12:3558. doi: 10.1038/s41467-021-23829-y (PMC8196201; doi:10.1038/s41467-021-23829-y)
Supplement: Supplementary file 1 — Supplementary Information [file 41467_2021_23829_MOESM1_ESM.pdf]

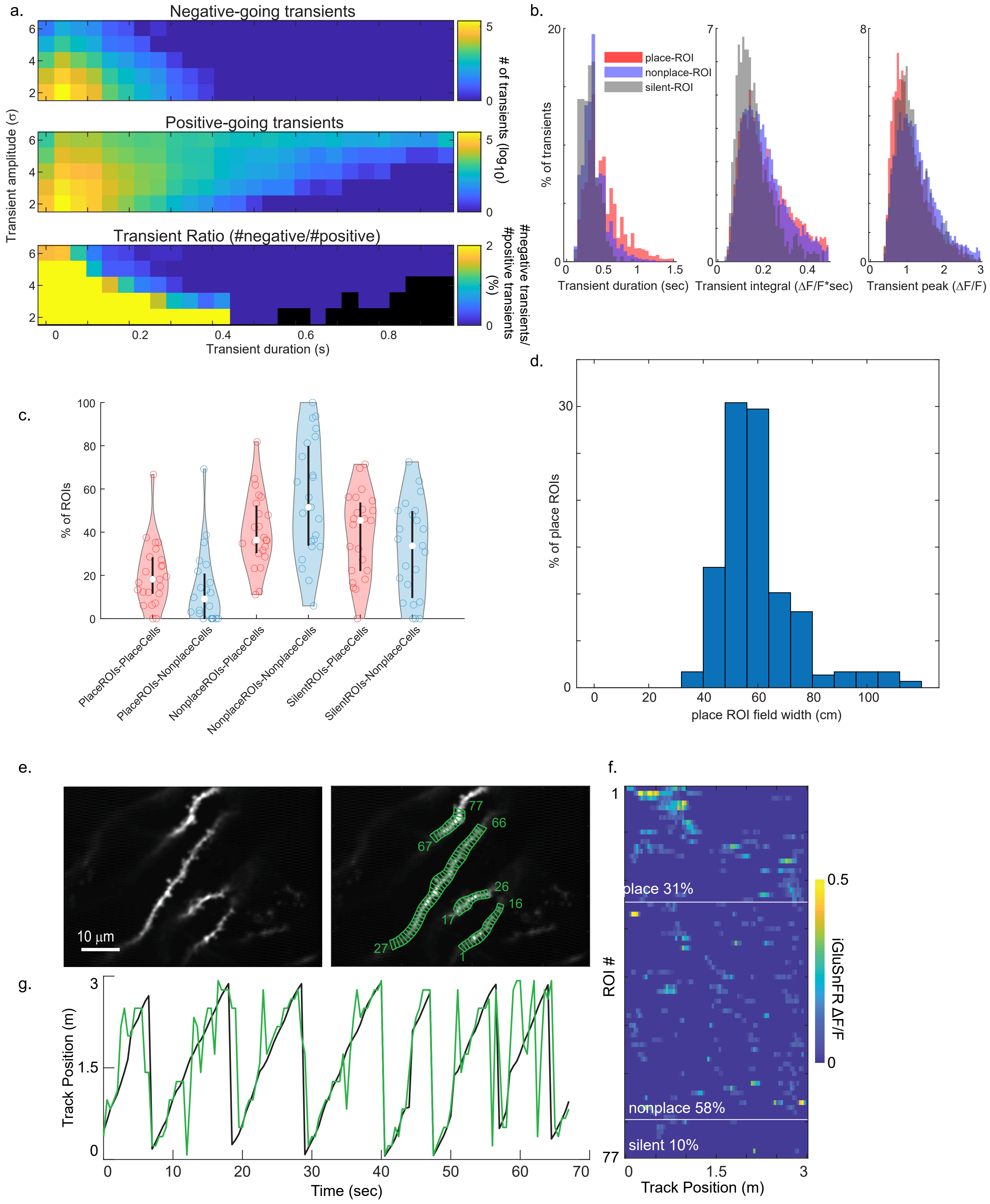

Supplementary Figure 1

Supplementary Figure 1: Characterization of properties of *in vivo* ROI iGluSnFR transients.

a. iGluSnFR transient statistics used to define significant transients from all iGluSnFR *in vivo* dendrite recordings, including place cells, nonplace cells, and dendrites that could not be traced to recorded somas. Top: Histogram of the number of negative going transients (noise and movement artifacts) of different durations and amplitudes (units of baseline standard deviation, σ). Middle: Histogram of the number of positive going candidate transients (functional transients, noise and movement artifacts) of different durations and amplitudes. Bottom: Ratio of number of negative to positive transients of different durations and amplitudes and converted to %. Significant transients are defined as transients of duration and amplitude for which the ratio of negative to positive transients is <0.01 (i.e $p<1\%$).

b. Histograms of iGluSnFR transient durations, integrals and peaks for place, active-nonplace and silent-ROIs from all cell types.

c. Percentage of ROIs in each ROI category for place vs nonplace cells calculated across cells (each circle represents the average percentage across the ROIs from a single cell). Note that with cell by cell averages, place cells have a greater percentage of place-ROIs than nonplace cells (Rank-sum $p= 0.039$, two-sided). Error bars and center of error bars represent first/third quartiles and medians, respectively.

d. Histogram of place-ROI field width (59.5 ± 14.5cm mean+/-SD) across all place-ROIs from all cells. n = 109 dendrites from 54 independent sessions from 11 mice.

e-g. Bayesian decoding of animal position by ROI transients.

e. Mean iGluSnFR image of 4 dendritic branches from the same cells at a single imaging plane (left). Right, same as left, but with 77 1-µm ROIs shown in green. Similar labeling results were obtained in 54 sessions from 11 mice.

f. Mean iGluSnFR $\Delta F/F$ versus track position across all traversals of a single session (mean ROI map) for all ROIs (each row represents a single ROI mean $\Delta F/F$) shown in **e** (right). Percentage of ROIs in each ROI category also shown. Plotted via cross-validation within each ROI category.

g. Mouse track position (black) and decoded position (green) using iGluSnFR transients and mean ROI maps from all ROIs shown in **e** (right). Average position error: 11.2%.

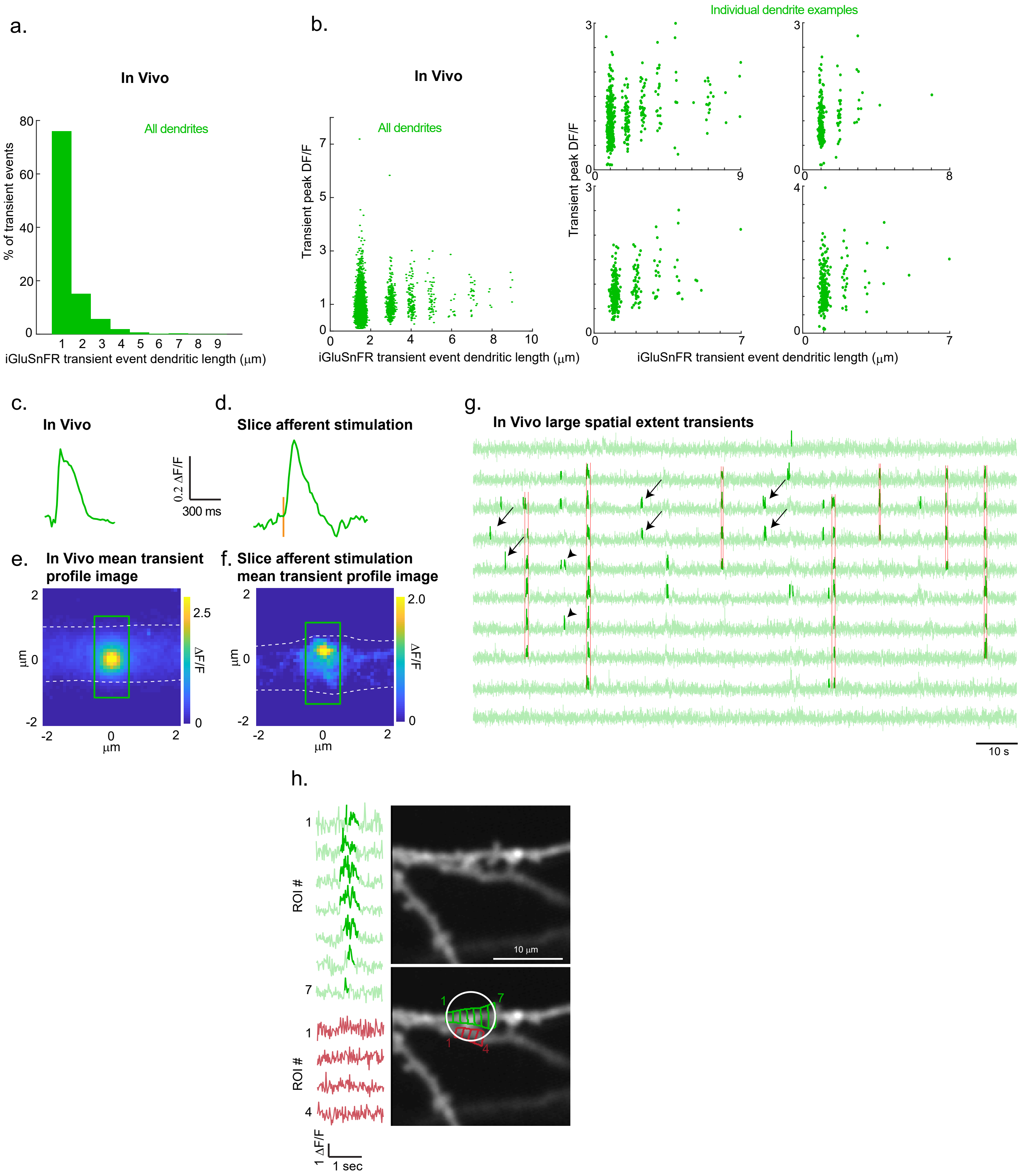

Supplementary Figure 2

Supplementary Figure 2: Characterization of spatiotemporal properties of single ROI and large spatial extent iGluSnFR transients.

a. Histogram of iGluSnFR transient event dendritic length measured in number of 1μm ROIs. Transients are from all iGluSnFR *in vivo* dendrite recordings, including place cells, nonplace cells, and dendrites that could not be traced to recorded somas.

b. Plot of peak ΔF/F amplitude versus iGluSnFR transient event dendritic length for all transients shown in **a** (left) and for four individual dendrite examples (right). Note that little or no correlation exists between the amplitude and spatial extent of the *in vivo* transients. Also note that in **a** most (76%) *in vivo* iGluSnFR transients were contained within a single 1-μm ROI and the amplitude of these transients often exceeded that of larger spatial extent transients (**b**). Further **a** and **b** show that the *in vivo* iGluSnFR transient peak amplitude was not related to the spatial extent of the transient, therefore larger glutamate release onto a single ROI does not necessarily lead to spillover over a larger dendritic distance. This finding is inconsistent with the hypothesis that large amounts of release per terminal or a large number of local coactive terminals (within a single 1-μm ROI) might lead to large amounts of glutamate that spills over and is detected on the dendrite over 4-10 μm. However, this finding is consistent with synchronized-release of glutamate from different presynaptic terminals onto multiple adjacent ROIs, our proposed mechanism to explain large spatial extent transients.

c. Triggered average of smallest 40% ΔF/F and shortest 85% duration *in vivo* iGluSnFR significant transients restricted to 1 μm ROIs (214 transients). Average triggered on transient onset.

d. Triggered average of slice iGluSnFR ΔF/F transients from all afferent stimulation successes (122; same as shown in Figure S3**f**). Average triggered on stimulation onset.

e. Mean image of iGluSnFR transients *in vivo* restricted to 1 μm ROIs. The full width at half maximum of 0.8 μm (Gaussian fit) suggests that most glutamate spillover beyond a synapse that is detected by iGluSnFR is highly localized (<~1 μm) on CA1 dendrites during behavior. Mean dendrite outlined in white dashed line. Size of 1 μm ROI (along length of dendrite) shown for reference in green.

f. Mean image of slice iGluSnFR ΔF/F transients from all afferent stimulation successes (same as shown in Figure S3**g** (top)). The full width at half maximum of 0.8 μm (Gaussian fit) for the success transients suggests that most glutamate spillover beyond a synapse that is detected by iGluSnFR is highly localized (<~1 μm) on CA1 dendrites during synaptic transmission. Mean dendrite outlined in white dashed line. Size of 1 μm ROIs (along length of dendrite) shown for reference in green. **c-f.** Note the similarity (amplitude, duration, spatial extent) between the average of the smallest *in vivo* transients and the transients from afferent stimulation in slice, suggesting that the smallest *in vivo* transients were likely generated from activation of a single synapse.

g. iGluSnFR ΔF/F vs time traces from ROIs on a single branch acquired during linear track navigation. Significant transients highlighted in bold green, and large spatial extent transients outlined in red. Note that the co-active adjacent ROIs recruited during the large spatial extent transients (red) could be recruited with fewer (arrows) and/or non-adjacent ROIs at other times (arrowheads),within just a few 10s of seconds of the large extent transients. This rapid variability is consistent with synchronized-release of glutamate from different presynaptic terminals onto multiple adjacent ROIs as the mechanism for large spatial extent transients.

h. Example large spatial extent transient observed *in vivo* over 7 neighboring ROIs in one dendritic branch (green), but not on a different branch that was <3.5 μm from the center of the active branch (red). White circle is 3.5 μm in radius and centered on the middle of the large spatial extent transient. ΔF/F iGluSnFR vs time traces seen at left for ROIs labeled in image at right, significant transients in bold (no detectable significant transients in red ROIs). We found 8 examples (across n = 109 dendrites from 54 sessions from 11 mice), such as the one shown here, with two nearby iGluSnFR labeled dendritic branches when a large spatial extent transient was observed in only one of the branches; importantly, we did not find any counter-examples (i.e. cross-talk between large spatial extent transients across closely opposed branches). Thus the glutamate release associated with large spatial extent transients is confined along the length of the dendrites, which is consistent with synchronized-release of glutamate from different presynaptic terminals onto

multiple adjacent ROIs as the mechanism for large spatial extent transients, but is not consistent with radially spreading spillover of glutamate from a central release site.

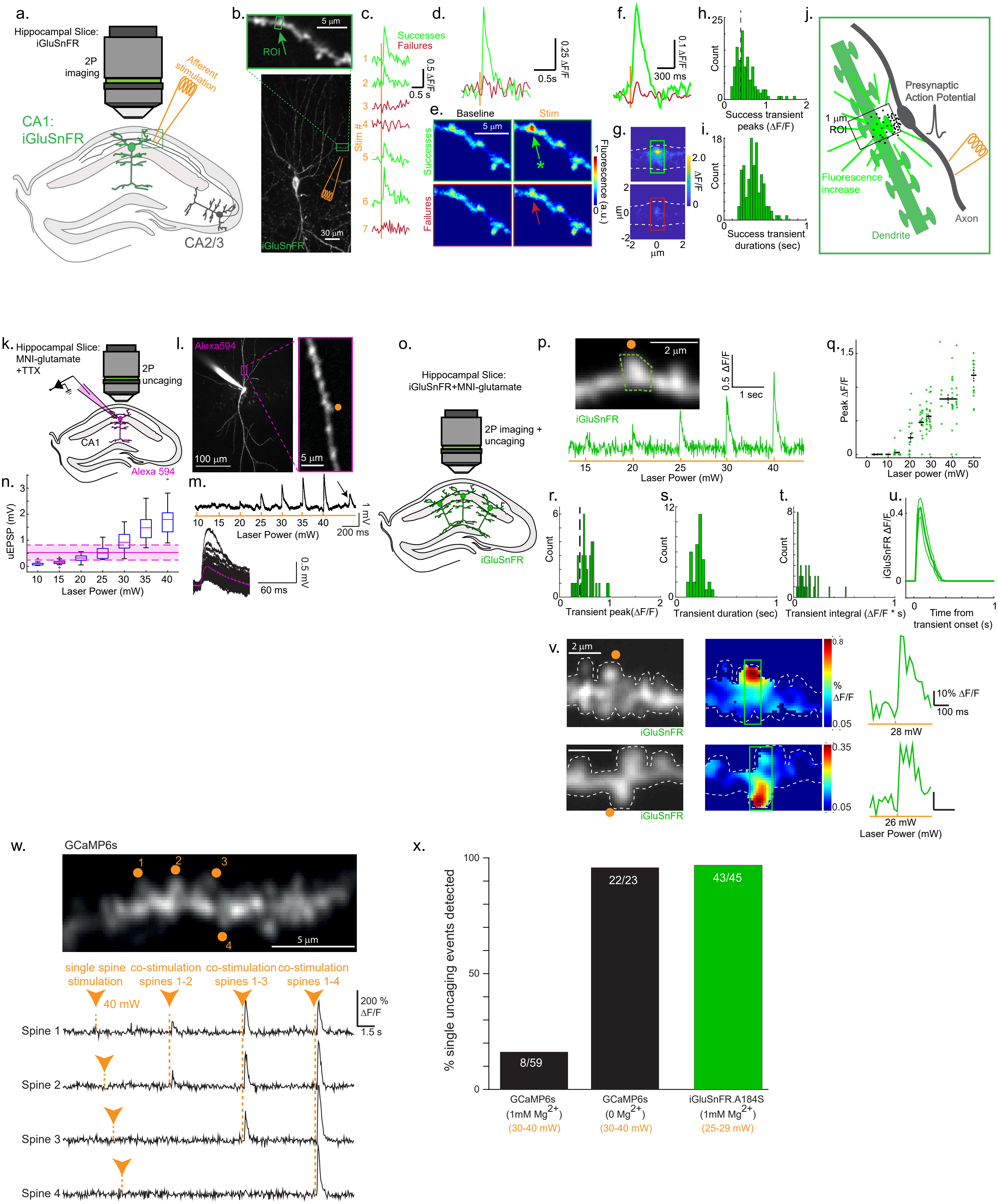

Supplementary Figure 3

Supplementary Figure 3: Slice characterization of iGluSnFR (and GCaMP6s) using axonal stimulation and glutamate uncaging.
a. Schematic depicting slice experiments with 2P iGluSnFR imaging combined with electrical stimulation of Schaffer collateral afferents. Stimulation was applied near dendrites of CA1 pyramidal neurons expressing iGluSnFR in a sparsely labeled CA1 population.
b. bottom, CA1 cell labeled with iGluSnFR with approximate position of stimulating electrode; top, dendritic segment imaged during stimulation with 1 μm ROI drawn around responsive synapse. Similar labeling results were found in slices from 6 mice.
c. Single trial ΔF/F vs time traces from ROI in **b** during seven successive single stimulations. Note the all-or-none nature of the iGluSnFR transients with glutamate release successes (statistically significant transients) time-locked to stimulation—green, and stimulation failures (no statistically significant transients)—red.
d. ΔF/F vs time stimulus triggered averages of successes and failures from **c**.
e. Average iGluSnFR fluorescence images before (Baseline) and after (Stim) stimulations for the successes and failures from **c**. Note the ~1 μm size increase in fluorescence observed during the successes (green arrow) and lack of increase observed during the failures (red arrow).
f. ΔF/F vs time stimulus triggered averages of successes (122) and failures (510) from all 59 ROIs from all recordings (n=6 mice).
g. Mean images of iGluSnFR ΔF/F for all 122 successes (top) and 510 failures (bottom). The full width at half maximum of 0.8 μm (Gaussian fit) for the success transients suggests that most glutamate spillover beyond a synapse that is detected by iGluSnFR is highly localized (<~1 μm) on CA1 dendrites during synaptic transmission. Mean dendrite outlined in white dashed line. Size of 1 μm ROIs (along length of dendrite) shown for reference in red and green. For the successes (top) the mean image is representative of the spatial spread observed in the 122 individual transients. These 122 transients were restricted to a 1 μm dendritic region, nearly all were encompassed in a single 1 μm ROI, but some were present in two adjacent 1 μm ROIs when the transient was bisected by the ROIs.
h-i. Histograms of peaks (**h**) and durations (**i**) of all 122 iGluSnFR fluorescence transient successes (in 1-μm ROIs). Amplitude cutoff threshold for significant iGluSnFR transients determined *in vivo* during behavior shown by dashed line (**h**). n = 59 ROIs from 6 independent mice.
j. Schematic model of iGluSnFR transient success generation from synaptic glutamate release during afferent stimulation.
k. Schematic depicting slice intracellular current clamp recording and 2P glutamate uncaging experiments. Glutamate was uncaged at dendrites of CA1 neurons filled with Alexa-594 in bath solution containing MNI-glutamate and TTX.
l. Left, Example neuron filled with Alexa-594; right, dendritic uncaging site marked with orange dot. Similar labeling was achieved in 6 slices from 4 mice.
m. Top, example membrane potential trace of uncaging evoked EPSPs (uEPSPs) driven using different laser powers from example shown in **l**. Spontaneous EPSP (arrow) in presence of TTX (miniature EPSP). Bottom, all miniature EPSPs from all recordings (black) and the average of all miniature EPSPs (purple). The rate of spontaneous EPSPs in the presence of TTX that we found (0.519 ±0.31 EPSPs/second) is in the range that has been previously reported: 0.5–1 Hz [Groc, Gustafsson, Hanse, J. Neurosci (2002)] to 1-2Hz [Hsia, Malenka, Nicoll, J. Neurophysiology (1998)].
n. Plot of mean peak membrane potential amplitude of uEPSPs vs. uncaging power from all recordings (16 uncaging sites, 11 branches, 6 slices, n = 4 mice). Mean miniature EPSP peak amplitude (solid purple line) and SD (shaded region out to dashed lines) from **m** shown directly overlapping uEPSPs from 25-29mW stimulations. Box minima and maxima represent 25th and 75th percentiles, box center represents median, whiskers extend to the most extreme data points not considered outliers, outliers are plotted with '+'.
o. Schematic depicting slice experiments with 2P iGluSnFR imaging combined with 2P glutamate uncaging. Glutamate was uncaged at dendrites of CA1 pyramidal neurons expressing iGluSnFR in a sparsely labeled CA1 population.
p. Top, Example dendritic segment from neuron expressing iGluSnFR; dendritic uncaging site marked with orange dot and 1-μm length ROI outlined in green. Bottom, example ΔF/F vs time trace during uncaging

with different laser powers from the example ROI and uncaging site shown at top. Similar labeling was found in 5 slices from 3 mice.

q. Plot of peak of iGluSnFR fluorescence transients (in 1 μm ROIs) vs. uncaging power from all recordings (29 uncaging sites, 14 branches, 5 slices, n = 3 mice)—green = individual transients, black = mean ± SE.

r-t. Histograms of peaks (mean 0.56±0.18 ΔF/F, **r**), durations (mean 230±60ms, **s**) and integrals (mean 0.058±0.003 (SD) ΔF/F*sec, **t**) of all iGluSnFR fluorescence transients (in 1-μm ROIs) generated using 25-29mW uncaging power. Amplitude cutoff threshold for significant iGluSnFR transients determined *in vivo* during behavior shown by dashed line. n = 29 uncaging sites from 14 independent branches in 5 slices from 3 mice.

u. Triggered average of all slice iGluSnFR transients generated by 25-29 mW stimulations, same transients as shown in **r-t**. Average triggered on transient onset.

v. Two examples of uncaging evoked iGluSnFR ΔF/F. Left, Mean iGluSnFR images; dendritic uncaging site marked with orange dots. Middle, iGluSnFR ΔF/F images from single uncaging stimuli. Dendrite outlined in white dashed line. 1 μm ROIs (along length of dendrite) shown in green. Right, ΔF/F vs time from green 1 μm ROIs from single uncaging stimuli.

w,x. By contrast, the detection sensitivity under the same conditions using a GECI (GCaMP6s,f) was far less, likely due to Mg2+ block of NMDA receptors. **w.** Top, Example dendritic segment from CA1 pyramidal neuron expressing GCaMP6s in hippocampal slice; dendritic uncaging sites marked with orange dots. Similar labeling was found in slices from 4 mice. Bottom, example ΔF/F vs time traces during uncaging using 40mW uncaging power (even larger than the 25-29 mW uncaging power used in **k-u** for iGluSnFR) from the example spines and uncaging sites shown at top (with 1mM Mg^{2+} in bath). No fluorescence transients were detectable when spines were individually stimulated (first stimulations, 300 ms inter-stimulus interval), but co-stimulation of 2 or more spines (120 μs inter-stimulus interval) led to detectable fluorescence transients—demonstrating that spines were responsive, and cooperativity was required in most cases to detect synaptic glutamate arrival with GCaMP6s. **x.** Percent of single uncaging events detected using GCaMP6s (with 1mM Mg^{2+} in bath; 30-40 mW; 14 branches, n = 4 mice), GCaMP6s (with 0mM Mg^{2+} in bath; 30-40 mW; 5 branches, n = 2 mice), or iGluSnFR (with 1mM Mg^{2+} in bath; 25-29 mW; 29 spines, 14 branches, n = 3 mice). While most events were not detected with GCaMP6s with Mg^{2+} in the bath, nearly all events were detected when Mg^{2+} was removed from the bath, suggesting that Mg^{2+} block of NMDA receptors prevents sufficient calcium influx to occur during uncaging to be detected by GCaMP6s.

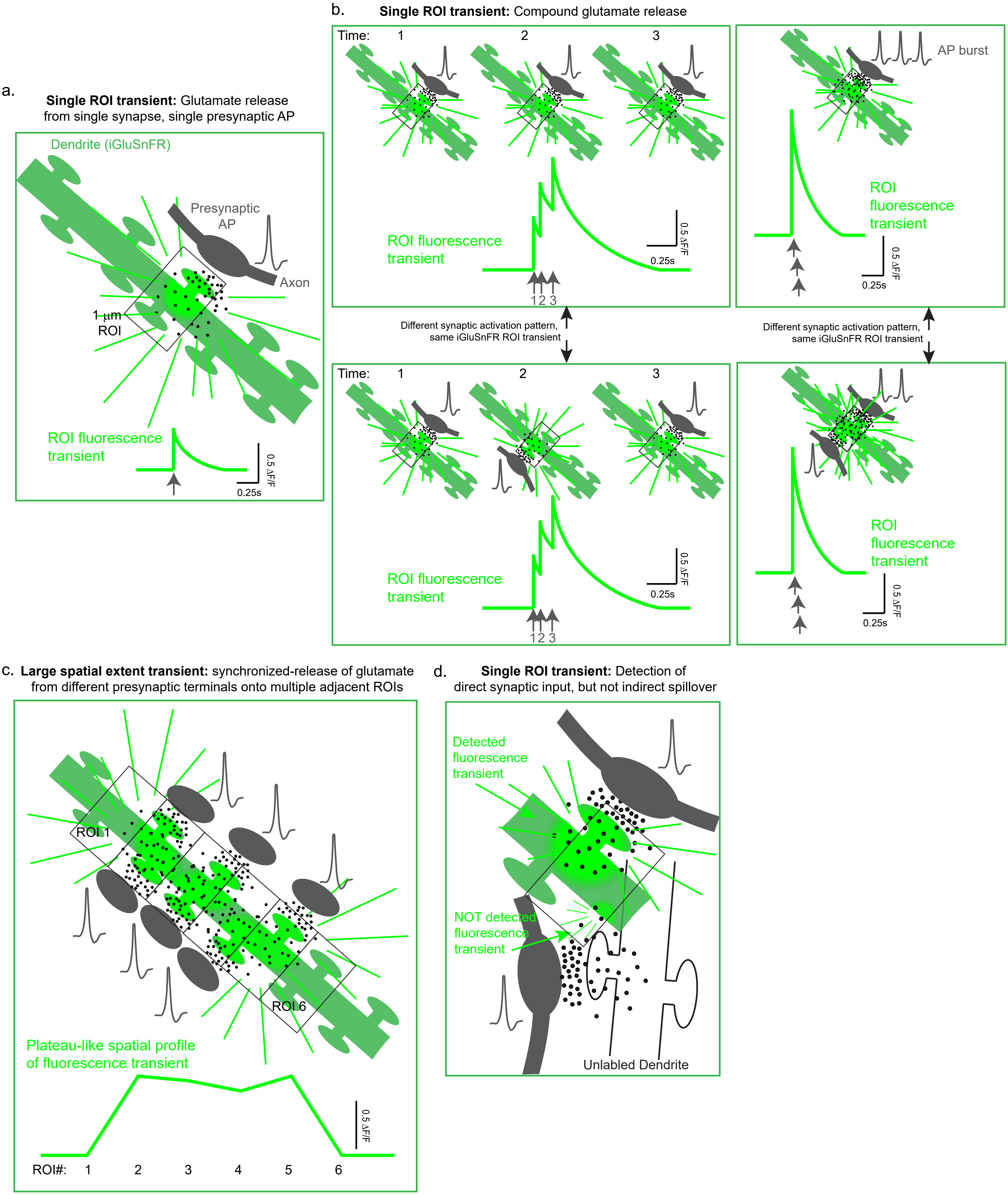

Supplementary Figure 4

Supplementary Figure 4: Schematic summary of the different spatiotemporal patterns of synaptic glutamate release presumably leading to the different amplitudes, durations and spatial extents observed for *in vivo* iGluSnFR transients.

a. Schematic model of iGluSnFR transient (from a 1-µm ROI) generated from synaptic glutamate release from a single presynaptic action potential (AP) at a single synapse.

b. Schematic model of iGluSnFR transient (from a 1-µm ROI) generated from different patterns of presynaptic activation: Left, multiple presynaptic action potentials spaced out in time (>~50ms) at a single synapse (top, left) or at different synapses (bottom, left) leading to multiple synaptic glutamate release events within the ROI within the decay time of iGluSnFR, and thus generating a compound transient of larger duration and amplitude than seen in **a**. Right, multiple presynaptic action potentials spaced out over a short time (<~10ms) at a single synapse (top, right) or at different synapses (bottom, right) leading to multiple synaptic glutamate release events within the ROI within the decay time of iGluSnFR, and thus generating a compound transient of larger amplitude than seen in **a**.

c. Schematic model of large spatial extent iGluSnFR transient generated from synchronized-release of glutamate (driven by presynaptic action potentials) from different presynaptic terminals onto multiple adjacent 1-µm ROIs. Profile of peak ΔF/F in each ROI shown at bottom; this plateau-like profile is consistent with most of the large spatial extent iGluSnFR transients observed in vivo (see Figure S5).

d. Schematic model of detectable change in iGluSnFR fluorescence from direct synapse, but undetectable change in iGluSnFR fluorescence from glutamate spillover from synapse onto unlabeled dendrite (i.e. little indirect spillover detection).

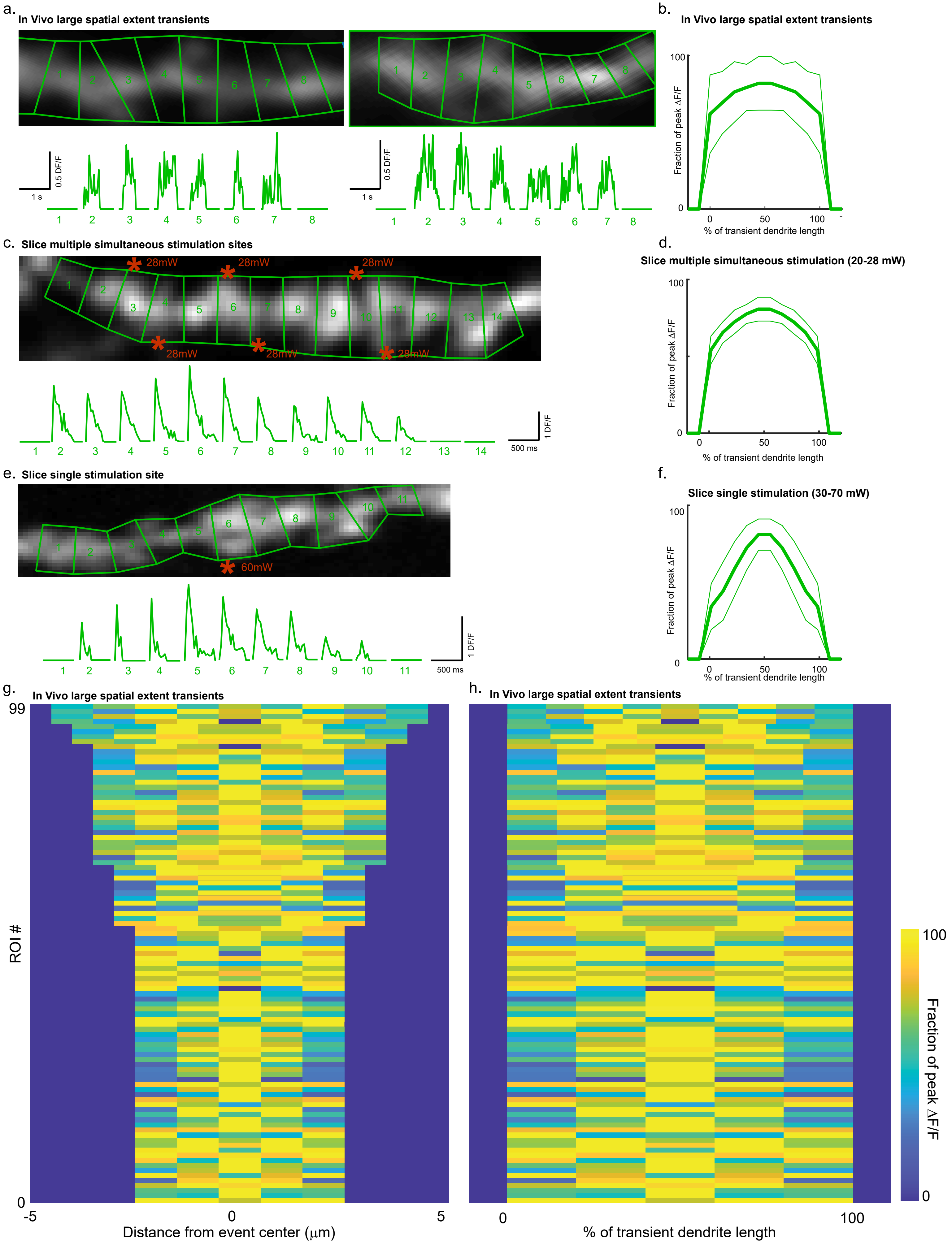

Supplementary Figure 5

Supplementary Figure 5: Characterization of spatial profile of large spatial extent iGluSnFR transients.
a. Two examples of *in vivo* large spatial extent iGluSnFR transients extending over 6 adjacent ROIs observed during spatial navigation. ROIs drawn in green on iGluSnFR labeled dendrite at top, and significant iGluSnFR transients from each ROI shown at bottom. Note the relatively flat (plateau-like) profile of the peak ΔF/F across the different ROIs containing the transients.
b. Mean profile of the peak ΔF/F across the different ROIs containing large spatial extent iGluSnFR transients, averaged over all large spatial extent transients >=5 ROIs in length observed *in vivo* during spatial navigation (99 transients, 11 mice). Note the relatively flat (plateau-like) profile, similar to the individual examples in **a.** Profiles from all 99 transients shown in **g** and **h** below.
c-f. We asked whether such a plateau-like profile seen in **a**,**b** could be generated by either synchronized-release of glutamate from different presynaptic terminals onto multiple adjacent ROIs, or synchronized -release of glutamate (from one or more terminals) onto a single ROI with glutamate spillover detected over adjacent ROIs. We mimicked these two different mechanisms in slice using glutamate uncaging and found that uncaging at multiple sites along a dendrite (**c,d**) resembled our plateau-like *in vivo* profile, whereas strong uncaging at a single site (**e,f**) could lead to spillover detection over 4-10 µm, but the profile was peaked and did not resemble our *in vivo* profile.
c. Example of large spatial extent iGluSnFR transient extending over 11 adjacent ROIs generated in slice using multi-site simultaneous glutamate uncaging. Uncaging sites (28 mW, 500 µs per site, 120 µs interstimulus site interval) marked with red asterisks at top, and significant iGluSnFR transients from each ROI shown at bottom. Note the relatively flat (plateau-like) profile of the peak ΔF/F across the different ROIs containing the transients.
d. Mean profile of the peak ΔF/F across the different ROIs containing large spatial extent iGluSnFR transients, averaged over all large spatial extent transients >=5 ROIs in length generated in slice using stimulation parameters detailed in **c** (23 transients, 2 mice, 25-29 mW). Note the relatively flat (plateau-like) profile, similar to the individual example in **c**, and importantly, similar to the mean *in vivo* profile shown in **b**.
e. Example of large spatial extent iGluSnFR transient extending over 9 adjacent ROIs generated in slice using a single large amplitude glutamate uncaging pulse. Uncaging site (60 mW, 500 µs) marked with red asterisk at top, and significant iGluSnFR transients from each ROI shown at bottom. Note the peaked (mountain-like) profile of the peak ΔF/F across the different ROIs containing the transients.
f. Mean profile of the peak ΔF/F across the different ROIs containing large spatial extent iGluSnFR transients, averaged over all large spatial extent transients >=6 ROIs in length generated in slice using stimulation parameters detailed in **e** (9 transients, 2 mice, >30 mW). Note the peaked (mountain-like) profile similar to the individual example in **e**, and importantly, dissimilar to the mean *in vivo* profile shown in **b.**
g, h. Spatial ΔF/F profiles from all 99 large spatial extent transients used in **b**. Each transient is shown in a different row, with the different ROIs making up each large spatial extent iGluSnFR transients shown in different columns (plotted as a function of distance in units of either microns (**g**) or % of transient length (**h**)). Peak ΔF/F in each ROI is indicated by the colormap.
For quantification (**g**, **h**), we examined the rate of ΔF/F change as a function of ROI distance from the center of each of the 99 events (i.e. the average slope of the spatial profiles). We then randomized the order of the ROIs in each large spatial extent transient and recalculated the slopes to generate a shuffle distribution. Importantly, 89 out of 99 transients had slopes within the 95% confidence bounds of the shuffle distribution, while only 11 displayed ΔF/F profiles with falloff greater than expected by chance. Therefore, the vast majority of the in vivo large spatial extent transients had profiles consistent with synchronized-release of glutamate from different presynaptic terminals onto multiple adjacent ROIs, with essentially randomized ΔF/F amplitude in the ROIs along the length of the event, leading to an average plateau-like profile. This result is inconsistent with a decreasing concentration of glutamate from a central release location, which would be expected to generate a more peaked profile and large spatial extent transients with consistently decreasing slopes (with values consistently less than the shuffle distribution).

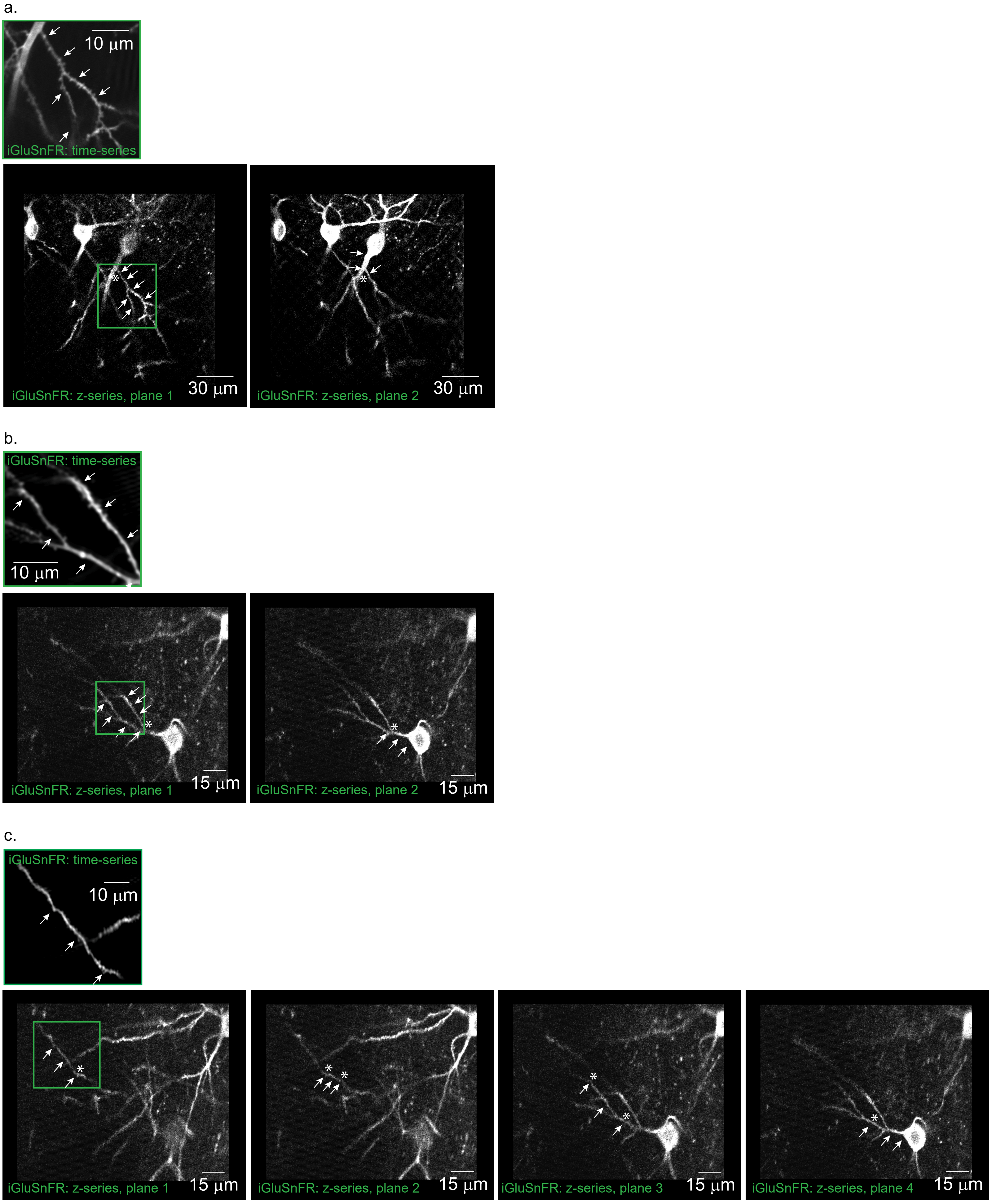

Supplementary Figure 6

Supplementary Figure 6: Tracing imaged dendrites back to parent soma with z-series.
a-c. Three representative examples showing the iGluSnFR time-series imaging plane (outlined in green, arrows indicate analyzed branches), the same imaging plane in the z-series (plane 1; green box: time-series imaging region; arrows indicate analyzed branches), and different focal planes in the z-series (planes >=2; arrows trace the imaged dendrite back to the soma; stars mark the same location from one z-plane to the next) in which the dendrite(s) can be followed back to their parent soma. The examples in **b**,**c** are from the same cell and dendrites shown in Figure 2b,e. Similar results were found from 46 place and nonplace cells from 11 mice.

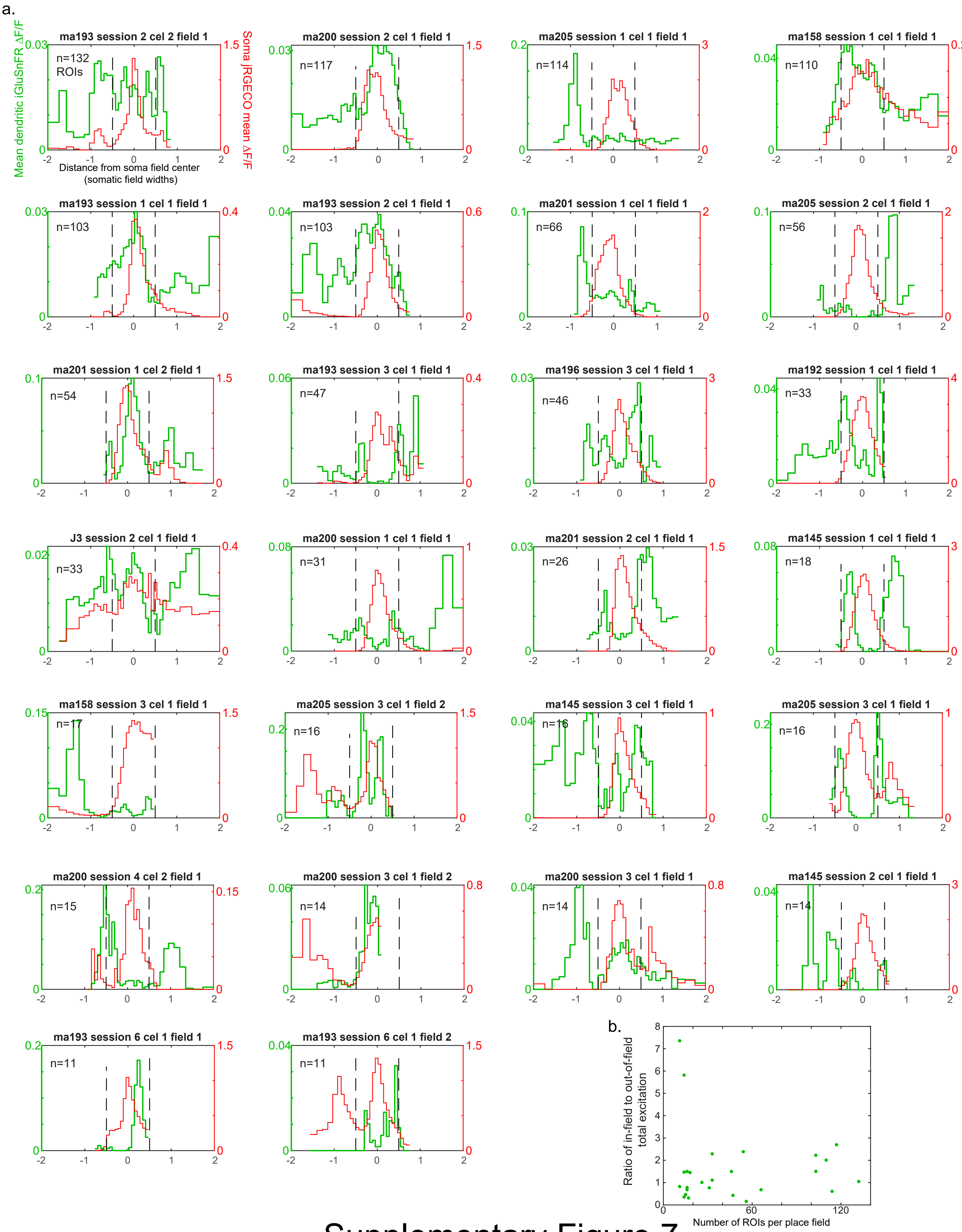

Supplementary Figure 7

Supplementary Figure 7: Individual place field examples of excitatory input versus somatic place field position.

a. Mean total excitatory input (green) as a function of distance from the center of the mean somatic place field (red) for all active ROIs plotted separately for each of the 26 different place fields (from 23 place cells). The average of these 26 traces was used to make the mean trace shown in Figure 3**b** (top). Interestingly, the iGluSnFR peak before the somatic field seen in Figure 3b (top and middle) is somewhat common as it can be seen in many different place fields: ma193 session 2 cell 2 field 1, ma205 session 1 cell 1 field 1, ma193 session 2 cell 1 field 1, ma201 session 1 cell 1 field 1, ma196 session 3 cell 1 field 1, ma145 session 3 cell 1 field 1, ma200 session 3 cell 1 field 1. Additionally, 16 out of the 26 place fields (~60%) had greater total excitatory input inside versus outside of the somatic place field.

b. The ratio of in-field to out-of-field total excitation for all 26 recordings (from 11 mice) shown in **a** versus the number of ROIs in each recording. No significant statistical correlation between this ratio and the number of ROIs was found (pearson's correlation: rho = -0.07, p=0.72).

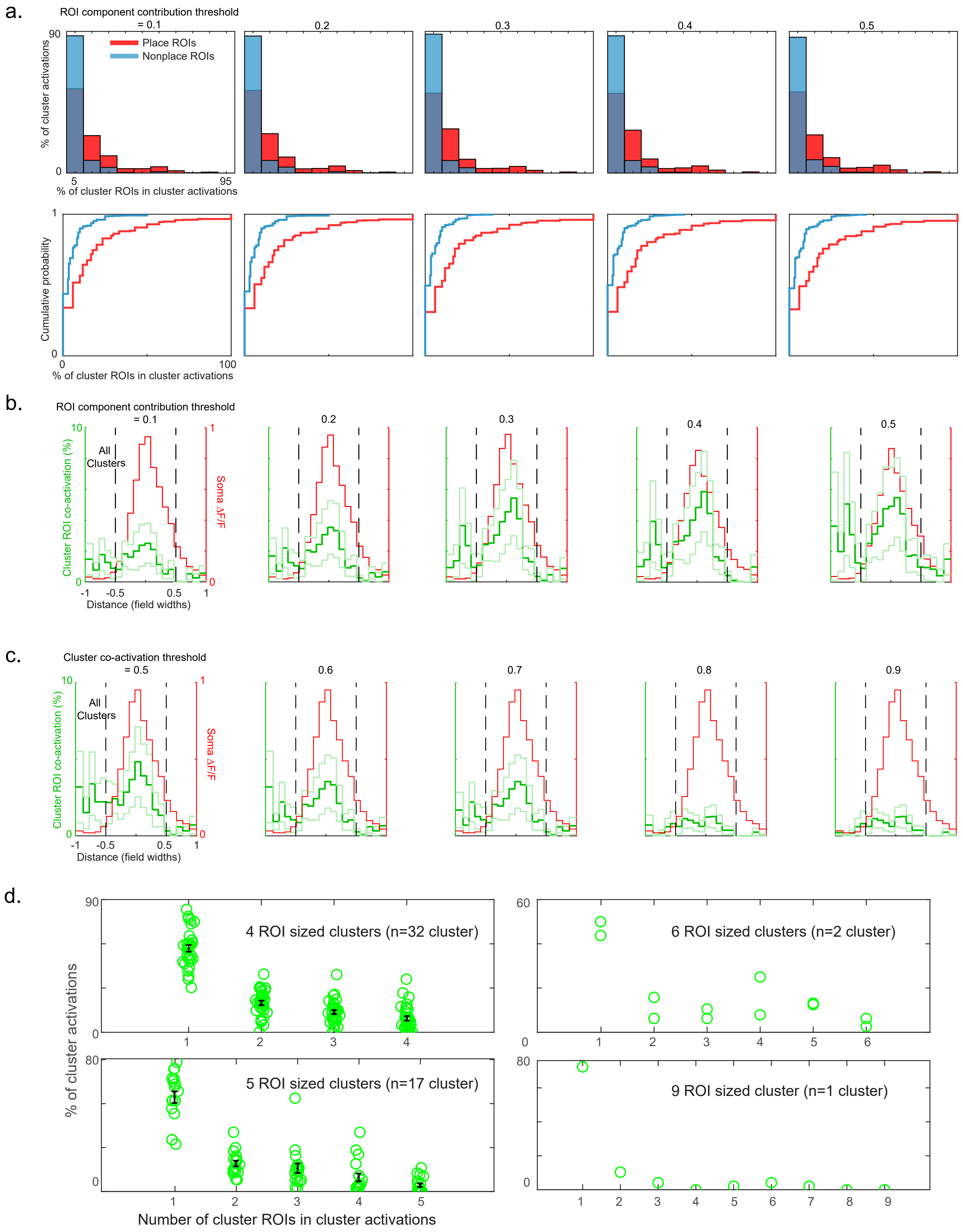

Supplementary Figure 8

Supplementary Figure 8: Cluster analysis is robust to changes in thresholds over a wide range of values.
a. Top, same as Figure 4**g**, but with different ROI component contribution thresholds. Bottom, same as Figure 4**h**, but with different ROI component contribution thresholds.
b. Same as Figure 4**i** (top), but with different ROI component contribution thresholds.
c. Same as Figure 4**i** (top), but with different cluster co-activation thresholds.
d. Histograms of the number of ROIs that were part of a cluster that were co-active during a cluster activation (non-zero cluster ΔF/F); histograms shown separately for different sized clusters ranging from 4-9 ROIs. Includes clusters from both place and nonplace cells. Circles represent individual clusters. Error bars represent SEM.

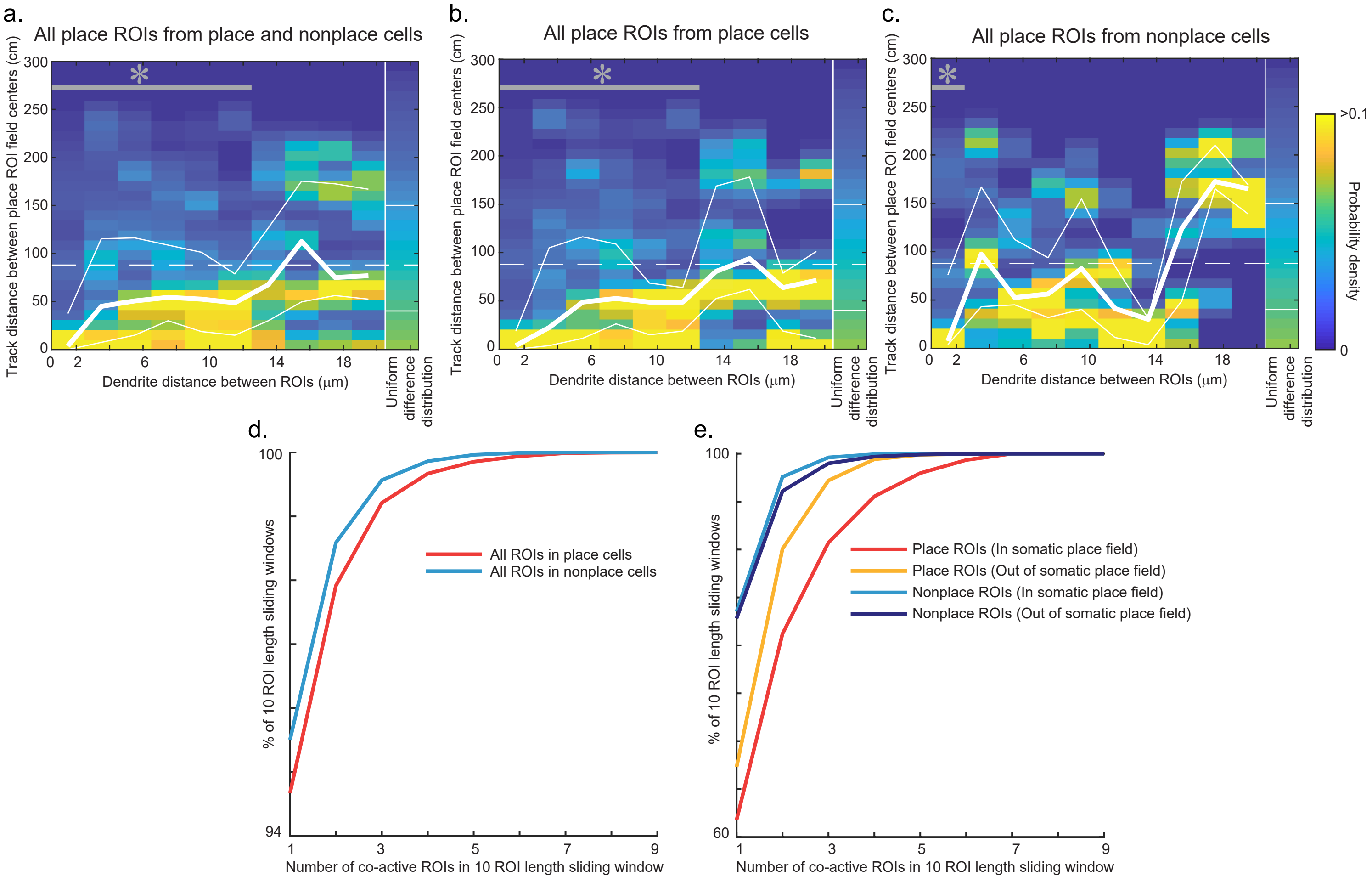

Supplementary Figure 9

Supplementary Figure 9: Further characterization of functional clustering of ROIs in place cells and place fields.

a. Track distance between place-ROI field centers (distance between COM of mean ΔF/F maps) of all pairs of place-ROIs on a single branch versus the dendritic distance between the pairs of ROIs, averaged over all branches from place and nonplace cells. Colors indicate probability density of ROI pairs (normalized in each column separately) and white lines indicate median ± 1 quartile of track distance vs dendrite distance. Grey line indicates dendritic distances with track distance distributions significantly different (Kolmogorov-Smirnov test, two-sided $p<0.0025$, Bonferroni corrected $alpha<0.05$) from uniform difference distribution shown in last column, with median track distance from uniform difference distribution shown with white dashed line.

b. Same as **a**, but for all pairs of place-ROIs from place cells.

c. Same as **a**, but for all pairs of place-ROIs from nonplace cells.

d. Cumulative probability from histograms of number of co-active ROIs in 10 ROI length sliding windows (see Methods) from place (red) or nonplace (blue) cells; $*p\sim0$ Rank-sum test, two-sided.

e. Cumulative probability from histograms of number of co-active ROIs in 10 ROI length sliding windows (see Methods) from place-ROIs or nonplace-ROIs, with sliding windows either inside or outside of somatic place field.

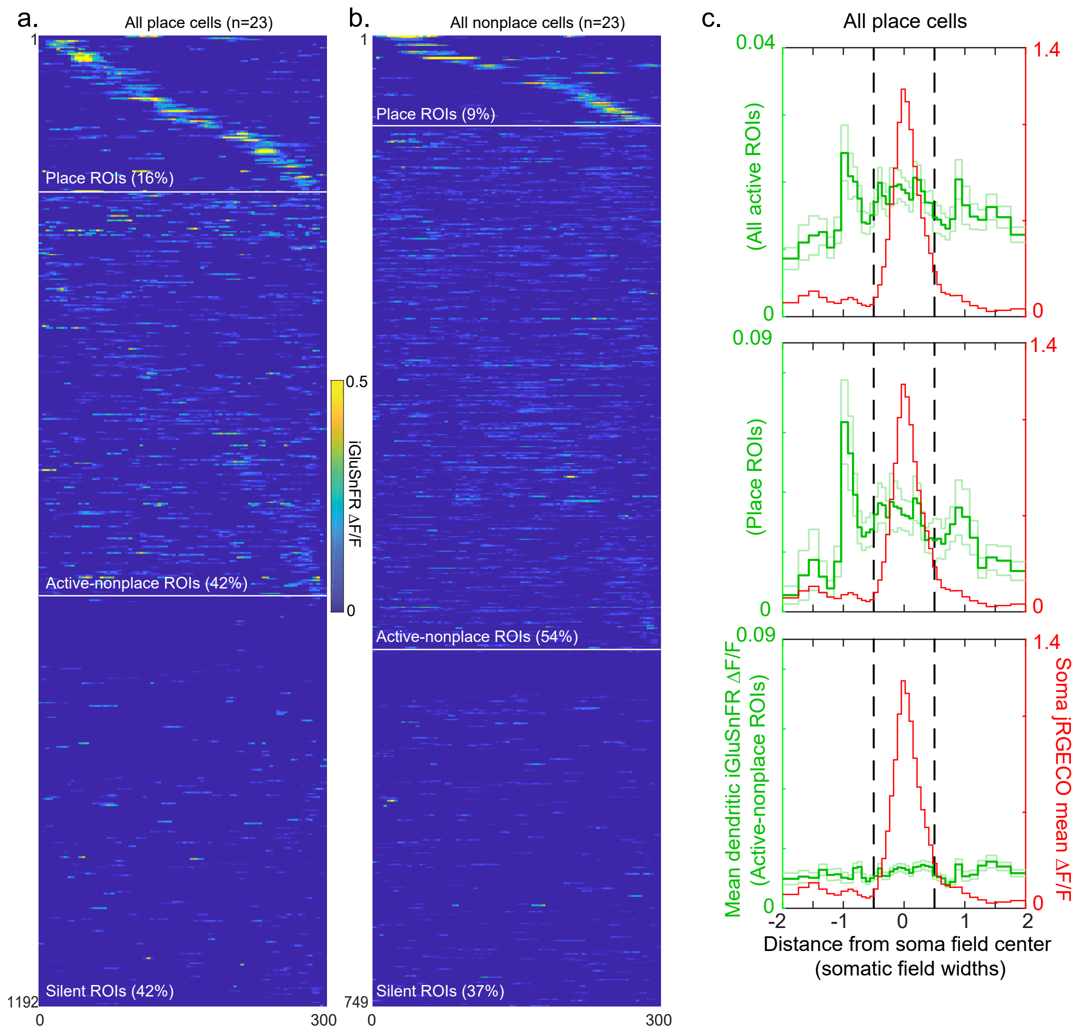

Supplemental Figure 10

Supplementary Figure 10: Large spatial extent transients do not significantly contribute to main results from Figures 2 and 3.
a. Same as Figure 2h, but with large spatial extent transients removed from analysis (large spatial extent transients were set to 0 in significant transient only traces for each contributing ROI; see Methods).
b. Same as Figure 2i, but with large spatial extent transients removed from analysis.
c. Same as Figure 3b, but with large spatial extent transients removed from analysis.
