# Supplementary material for: The functional organization of excitatory synaptic input to place cells
Source: Nat Commun. 2021 Jun 11;12:3558. doi: 10.1038/s41467-021-23829-y (PMC8196201; doi:10.1038/s41467-021-23829-y)
Supplement: Supplementary file 3 — Reporting Summary [file 41467_2021_23829_MOESM3_ESM.pdf]

nature research

Corresponding author(s): Daniel Dombeck

Last updated by author(s): 4-7-2021

Reporting Summary

Nature Research wishes to improve the reproducibility of the work that we publish. This form provides structure for consistency and transparency in reporting. For further information on Nature Research policies, see our Editorial Policies and the Editorial Policy Checklist.

Statistics

For all statistical analyses, confirm that the following items are present in the figure legend, table legend, main text, or Methods section.

n/a	Confirmed	
☐	☒	The exact sample size (*n*) for each experimental group/condition, given as a discrete number and unit of measurement
☐	☒	A statement on whether measurements were taken from distinct samples or whether the same sample was measured repeatedly
☐	☒	The statistical test(s) used AND whether they are one- or two-sided<br>*Only common tests should be described solely by name; describe more complex techniques in the Methods section.*
☐	☒	A description of all covariates tested
☐	☒	A description of any assumptions or corrections, such as tests of normality and adjustment for multiple comparisons
☐	☒	A full description of the statistical parameters including central tendency (e.g. means) or other basic estimates (e.g. regression coefficient) AND variation (e.g. standard deviation) or associated estimates of uncertainty (e.g. confidence intervals)
☐	☒	For null hypothesis testing, the test statistic (e.g. F, t, r) with confidence intervals, effect sizes, degrees of freedom and P value noted<br>*Give P values as exact values whenever suitable.*
☒	☐	For Bayesian analysis, information on the choice of priors and Markov chain Monte Carlo settings
☒	☐	For hierarchical and complex designs, identification of the appropriate level for tests and full reporting of outcomes
☒	☐	Estimates of effect sizes (e.g. Cohen's *d*, Pearson's *r*), indicating how they were calculated

Our web collection on statistics for biologists contains articles on many of the points above.

Software and code

Policy information about availability of computer code

Data collection	Matlab r2018a, Vidrio technologies ScanImage 2017, Molecular Devices Clampex 10.3, Labview 2011
Data analysis	ImageJ (Version 1.47), custom software written in MATLAB (r2018a). The custom code that support the findings of this study are available from the corresponding author upon reasonable request.

For manuscripts utilizing custom algorithms or software that are central to the research but not yet described in published literature, software must be made available to editors and reviewers. We strongly encourage code deposition in a community repository (e.g. GitHub). See the Nature Research guidelines for submitting code & software for further information.

Data

Policy information about availability of data

All manuscripts must include a data availability statement. This statement should provide the following information, where applicable:

- Accession codes, unique identifiers, or web links for publicly available datasets
- A list of figures that have associated raw data
- A description of any restrictions on data availability

The data that support the findings of this study are available from the corresponding author upon reasonable request.

Field-specific reporting

Life sciences study design

All studies must disclose on these points even when the disclosure is negative.

Sample size	No statistical methods were used to predetermine sample sizes. Sample sizes were based on reliably measuring experimental parameters while remaining in compliance with ethical guidelines to minimize the number of animals used. Kolmogorov-Smirnov tests, T tests, Rank Sum tests, Shuffle tests, Likelihood ratio tests, and Wilcoxon rank tests were used to test for statistical significance when appropriate and all statistical tests were two-sided unless stated otherwise.
Data exclusions	All inclusion/exclusion criteria were established independently and prior to analysis of effect sizes. For imaging experiments (iGluSnFR and GFP control), after motion correction the time-series were visually inspected and either included if in plane movements were small in comparison to the structures of interest (width of the dendrites) and out of plane (z) movement was minimal, or excluded if in plane movements were larger than the structures of interest or if out of plane movement was visible. These criteria were strictly applied and were biased towards rejecting any borderline cases; ~2/3 of acquired dendritic time-series were able to be sufficiently motion corrected and included for further analysis. Thus, our inclusion criteria only focused on the structure (movement) of the dendritic branches, and did not include any functional iGluSnFR (or GFP) measures.<br>ROIs with mean fluorescence of <1.5 counts were too dim for an accurate measure of ΔF/F and were excluded from further analysis; these criteria excluded 8.6% of ROIs. Time periods (frames) in which large out of plane movements were observed across many ROIs were excluded from further analysis; these periods were identified by calculating the mean fluorescence versus time trace across all ROIs in a field, calculating the STD of this trace, and then excluding any time periods in which the fluorescence was >2STD from the mean in the positive direction or >1STD from the mean in the negative direction; this excluded 0.3% of frames during track running.
Replication	Experimental results were based on neural activity patterns and mouse behavior during tasks. Multiple mice (11 for iGluSnFR experiments and 4 for GFP controls) were used for every experiment and observations of similar results across mice were used to infer replication. All attempts at replication were successful.
Randomization	Randomization was not required because this study did not allocate different mouse experimental groups.
Blinding	Blinding was not required because this study did not allocate different mouse experimental groups.

Reporting for specific materials, systems and methods

We require information from authors about some types of materials, experimental systems and methods used in many studies. Here, indicate whether each material, system or method listed is relevant to your study. If you are not sure if a list item applies to your research, read the appropriate section before selecting a response.

Materials & experimental systems

n/a	Involved in the study	
☒	☐	Antibodies
☒	☐	Eukaryotic cell lines
☒	☐	Palaeontology and archaeology
☐	☒	Animals and other organisms
☒	☐	Human research participants
☒	☐	Clinical data
☒	☐	Dual use research of concern

Methods

n/a	Involved in the study	
☒	☐	ChIP-seq
☒	☐	Flow cytometry
☒	☐	MRI-based neuroimaging

Animals and other organisms

Policy information about studies involving animals; ARRIVE guidelines recommended for reporting animal research

Laboratory animals	9 to 12 weeks old male C57BL/6J (WT, Charles River) mice (20-30 g) were individually housed under a reverse 12 hr light/dark cycle, in 40-60% humidity at 65-75° F.
Wild animals	This study did not involve wild animals.
Field-collected samples	This study did not involve samples collected from the field.
Ethics oversight	Northwestern University Animal Care and Use Committee

Note that full information on the approval of the study protocol must also be provided in the manuscript.
