# Supplementary material for: The functional organization of excitatory synaptic input to place cells
Source: Nat Commun. 2021 Jun 11;12:3558. doi: 10.1038/s41467-021-23829-y (PMC8196201; doi:10.1038/s41467-021-23829-y)
Supplement: Supplementary file 2 — Peer Review File [file 41467_2021_23829_MOESM2_ESM.pdf]

Editorial Note: This manuscript has been previously reviewed at another journal that is not operating a transparent peer review scheme. This document only contains reviewer comments and rebuttal letters for versions considered at Nature Communications. Mentions of the other journal have been redacted.

Reviewers' Comments:

Reviewer #1:
Remarks to the Author:
In this revision of a paper that I originally reviewed for [redacted], the authors have addressed all of my initial concerns. I felt that the original submission was quite strong and I am enthusiastic about the manuscript in its current form.

Reviewer #3:
Remarks to the Author:
The authors have very carefully revised heir manuscript. The changes reflect the nuances and details of this work much better than the previous version. In the current format the authors claims are nicely supported by the data presented.
The authors have satisfactorily addressed all the issues I erased previously, I found the changes they made to be appropriate.

My opinion on the novelty of the study did not change, I remain positive about this aspect of the study. My concerns were largely focused on data interpretation and the validity of some of the claims the author made. In their careful rebuttal they addressed all these issues.

Reviewer #1 (Remarks to the Author):

In this revision of a paper that I originally reviewed for [redacted], the authors have addressed all of my initial concerns. I felt that the original submission was quite strong and I am enthusiastic about the manuscript in its current form.

We thank the reviewer for their interest and support of our manuscript.

Reviewer #3 (Remarks to the Author):

The authors have very carefully revised heir manuscript. The changes reflect the nuances and details of this work much better than the previous version. In the current format the authors claims are nicely supported by the data presented.

The authors have satisfactorily addressed all the issues I erased previously, I found the changes they made to be appropriate.

My opinion on the novelty of the study did not change, I remain positive about this aspect of the study. My concerns were largely focused on data interpretation and the validity of some of the claims the author made. In their careful rebuttal they addressed all these issues.

We are happy that the reviewer appreciated our changes to the manuscript in response to their questions and concerns. We thank the reviewer for their interest and support of our manuscript.
